# *miR-34* Modulates Innate Immunity and Ecdysone Signaling in *Drosophila*

**DOI:** 10.1371/journal.ppat.1006034

**Published:** 2016-11-28

**Authors:** Xiao-Peng Xiong, Krishna Kurthkoti, Kung-Yen Chang, Jian-Liang Li, Xingjie Ren, Jian-Quan Ni, Tariq M. Rana, Rui Zhou

**Affiliations:** 1 Tumor Initiation and Maintenance Program; Sanford Burnham Prebys Medical Discovery Institute, California, United States of America; 2 Development, Aging and Regeneration Program, Sanford Burnham Prebys Medical Discovery Institute, California, United States of America; 3 Department of Pediatrics, University of California San Diego School of Medicine, California, United States of America; 4 Sanford Burnham Prebys Medical Discovery Institute, Orlando, Florida, United States of America; 5 Gene Regulatory Laboratory, School of Medicine, Tsinghua University, Beijing, China; INDIA

## Abstract

microRNAs are endogenous small regulatory RNAs that modulate myriad biological processes by repressing target gene expression in a sequence-specific manner. Here we show that the conserved miRNA *miR-34* regulates innate immunity and ecdysone signaling in *Drosophila*. *miR-34* over-expression activates antibacterial innate immunity signaling both in cultured cells and *in vivo*, and flies over-expressing *miR-34* display improved survival and pathogen clearance upon Gram-negative bacterial infection; whereas *miR-34* knockout animals are defective in antibacterial defense. In particular, *miR-34* achieves its immune-stimulatory function, at least in part, by repressing the two novel target genes *Dlg1* and *Eip75B*. In addition, our study reveals a mutual repression between *miR-34* expression and ecdysone signaling, and identifies *miR-34* as a node in the intricate interplay between ecdysone signaling and innate immunity. Lastly, we identify *cis*-regulatory genomic elements and *trans*-acting transcription factors required for optimal ecdysone-mediated repression of *miR-34*. Taken together, our study enriches the repertoire of immune-modulating miRNAs in animals, and provides new insights into the interplay between steroid hormone signaling and innate immunity.

## Author Summary

microRNAs are small regulatory RNAs that impact myriad biological processes. Here we show that the conserved miRNA *miR-34* regulates antibacterial defense and steroid hormone signaling in *Drosophila*. *miR-34* over-production or deficiency, respectively, enhances or impairs antibacterial defense. We show that *Dlg1* and *Eip75B* are two novel *miR-34* target genes relevant to innate immunity. In addition, our study reveals a mutual repression between *miR-34* and steroid hormone signaling and identifies genomic elements and transcription factors required for steroid hormone -mediated repression of *miR-34*. Thus our study identifies *miR-34* as a node linking steroid hormone signaling and immunity, thereby enriching the repertoire of immune-modulating miRNAs in animals and providing insights into the interplay between steroid hormone signaling and innate immunity.

## Introduction

Multi-cellular host organisms share the same environment with numerous microbes, and have developed robust defense mechanisms to combat invading microbial pathogens. The fruit fly *Drosophila melanogaster* relies exclusively on innate immunity, the first line of defense, to control microbial infections [1]. For example, upon systemic Gram-negative bacterial infection via septic injury, the *im*mune *d*eficiency (**IMD**) pathway is activated [2–6], which involves binding of diaminopimelic acid (**DAP**)-type peptidoglycan (**PGN**) present in Gram-negative bacteria by the trans-membrane *p*eptido*g*lycan-*r*ecognition *p*rotein (**PGRP-LCx**) and oligimerization of PGRP-LCx. This in turn, leads to the recruitment of the adaptor proteins Immune deficiency (IMD) and Fas associated death domain-containing protein (dFADD), and subsequent recruitment and activation of the Death related ced-3/Nedd2-like caspase Dredd. Next, Dredd cleaves IMD and the neo-C-terminal fragment of IMD binds to and activates the E3 ubiquitin ligase *Drosophila* Inhibitor of Apoptosis protein 2 (DIAP2), leading to poly-ubiquitination of IMD and Dredd, as well as the activation of the MAP3K TGF-β activated kinase 1 (dTAK1) and the D*rosophila* m*elanogaster Iκ*B *k*inase complex (**DmIKK**) [7–13], which phosphorylates the composite *Drosophila* NF-*κ*B protein Relish. In addition, Dredd carries out endoproteolytic cleavage of Relish [14–17]. The N-terminal fragment of Relish translocates to the nucleus and activates the transcription of genes encoding potent anti-bacterial peptides, such as *Diptericin* [18, 19]. In addition, in response to Gram-positive bacterial or fungal infection, the Toll pathway is activated, leading to nuclear translocation of another *Drosophila* NF-*κ*B family member Dorsal-related immunity factor (Dif) and activation of genes encoding potent anti-fungal and anti-bacterial peptides, such as *Drosomycin* [20–24]. Note that some Gram-positive bacteria (e.g. the *Bacillus* species) contain a DAP-type PGN, which is recognized by PGRP-LC [25], whereas PGRP-SD may participate in triggering Toll signaling [26, 27]. Lastly, in addition to the afore-mentioned systemic humoral immunity, which relies on AMPs, *Drosophila* also harbors cellular immunity, which are carried out by specialized hemocytes, including phagocytosis of invading microbes, melanization at the infection sites and encapsulation of larger invading objects such as parasitic eggs [28]. Cellular and humoral immunity work together and constitute a robust defense system that protects *Drosophila* from invading pathogens.

Ecdysone is a steroid hormone essential for *Drosophila* development. Ecdysone binds to the stereotypical steroid hormone receptor complex, a heterodimer composed of the *Ec*dysone *r*eceptor (**EcR**) protein and its co-factor Ultraspiracle, which functions as a transcription factor and modulates the expression of ecdysone target genes [29]. Ecdysone treatment triggers a rapid activation of a group of early response genes encoding transcription factors (referred to as *e*cdysone-*i*nduced *p*roteins or **EIP**s), which in turn regulate the expression of late ecdysone response genes. Ecdysone can profoundly alter the gene expression profile both in cultured Schneider (S2) cells and *in vivo*, thereby regulating various key aspects of *Drosophila* development and physiology, including innate immunity [30–32]. For example, it has been reported that ecdysone can activate the expression of *PGRP-LC* [33], thereby potentiating the IMD innate immunity signaling pathway both in cultured S2 cells and *in vivo*. In addition, ecdysone also strongly regulates the cellular immune response [34, 35].

On the one hand, effective control of pathogens depends on rapid and robust induction of the innate immune response; on the other hand, prolonged or aberrant activation of innate immunity signaling is detrimental to the host, and is associated with a number of pathological conditions in humans. For example, dysregulation of NF-*κ*B signaling contributes to autoimmunity and inflammatory diseases, and can cause several hematopoietic malignancies and various solid tumors [36]. Thus both the magnitude and the duration of innate immunity activation need to be tightly controlled at multiple stages. Genetic screening and gene expression profiling studies have led to the identification of a number of negative modulators of IMD signaling [37]. For example, the amidases peptidoglycan-recognition proteins PGRP-LB and PGRP-SC degrade Gram-negative bacteria peptidoglycan, thereby dampening IMD signaling [38, 39]. In addition, the *P*GRP-LC-interacting *i*nhibitor of *I*MD *s*ignaling (**PIMS**)/Pirk/Rudra associates with PGRP-LCx and IMD and causes the depletion of PGRP-LCx from the plasma membrane, thereby suppressing IMD signaling and facilitating to establish immune tolerance to commensal bacteria and maintain a balanced IMD response following oral and systemic infection [40–42]. Furthermore, Caspar, which is homologous to Fas-associating factor 1 in mammals, strongly prevents constitutive activation of IMD signaling by blocking Dredd-dependent nuclear translocation of Relish [43]. Lastly, additional negative regulators of the IMD pathway include the *Drosophila* homolog of the human cylindromatosis (CYLD) tumor suppressor, a de-ubiquitination enzyme, and the scaffold protein Plenty of SH3 (POSH), which probably operate to inhibit IKK and dTAK1, respectively [44, 45].

In addition to the afore-mentioned protein modulators of innate immunity signaling, non-coding regulatory RNAs such as *mi*cro*RNA*s (**miRNA**s) have also been implicated in immune regulation. miRNAs are a class of 22–24 nt endogenous regulatory small RNAs that function as key regulators of gene expression. miRNAs join the *miR*NA-*i*nduced *s*ilencing *c*omplexes (**miRISC**) and guide the miRISC to engage target mRNAs via complementary base-pairing between the seed region of miRNAs (positions 2–8) and miRNA-binding sites (primarily in the 3’ UTR of target mRNAs), leading to a reduction in protein output from target mRNAs by a combination of mRNA destabilization and/or translation inhibition [46–52]. A single mRNA can be targeted by multiple miRNAs. Conversely, each miRNA can potentially repress multiple mRNAs. It has been estimated that a significant fraction of mRNAs are subject to miRNA regulation [53].

miRNAs can profoundly impact the intensity and/or duration of immune signaling. For example, in mammals *miR-155* is highly induced during the macrophage inflammatory response and contributes to TNF*α* production [54, 55]. In addition, *miR-146a* is another mammalian miRNA induced by NF-*κ*B-signaling and controls Toll-like receptor and cytokine signaling by repressing TNF receptor-associated factor 6 and IL-1 receptor-associated kinase 1 genes [56]. Similarly, in *Drosophila* both *miR-8* and *let-7* have been implicated in fine-tuning IMD and/or Toll signaling [57–59]. However, it is clear that additional miRNAs that confer immune-modulating functions remain to be identified and functionally characterized. We screened a collection of 101 *Drosophila* miRNAs by examining the impact of their mis-expression *in vivo* on innate immunity signaling. This led to the identification of *miR-34* among several other miRNAs as modulators of IMD signaling. In particular, *miR-34* over-expression in cultured cells or *in vivo* causes aberrant activation of IMD signaling both in the absence and in the presence of immune challenge, and flies over-expressing *miR-34* display improved survival and pathogen clearance upon Gram-negative bacterial infection. In contrast, *miR-34* mutant flies present profound defects in antibacterial innate immunity. In addition, we found that the immune-modulating role of *miR-34* is critically dependent on IMD signaling, and that *miR-34* operates in part by repressing genes encoding the septate junction protein Dlg1 and the nuclear hormone family transcription factor Eip75B, a key mediator of the ecdysone steroid hormone signaling cascade. Furthermore, our analysis reveals that ecdysone strongly inhibits *miR-34* expression via transcriptional repression in a manner that is dependent on a number of transcription factors, including the ecdysone receptor and the *Br*oad *C*omplex (**BrC**), key mediators of ecdysone signaling cascade. Lastly, we identify ecdysone-responsive regulatory elements required for ecdysone-mediated repression of *miR-34* expression. Take together, our study identifies *miR-34* as a modulator of innate immunity, identifies both *cis*-regulatory elements and *trans*-acting transcription factors required for ecdysone-mediated repression of *miR-34*, and reveals that the cross-regulation between ecdysone signaling and *miR-34* expression contributes to optimal levels of immune activation upon microbial challenge.

## Results

### Dysregulation of miRNA biogenesis impacts innate immunity signaling

**Fig 1:**
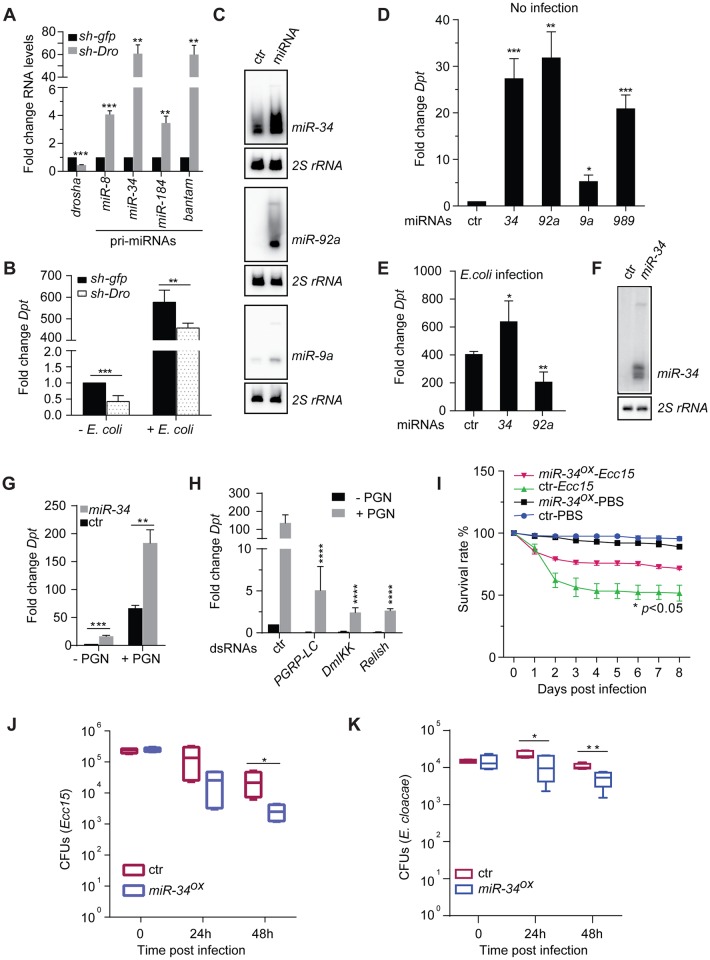
Over-expression of *miR-34* activates innate immunity signaling. (**A**) Total RNA was isolated from male progeny from crossing flies carrying the ubiquitously expressed *da-Gal4* driver and a temperature-sensitive *Gal80* transgene (*da>Gal4 tub-Gal80* ^*ts*^) to UAS-shRNA lines targeting *Drosha* or the control *gfp*. Flies crosses were kept at 18°C and progeny were shifted to 29°C for 5 days upon eclosure to induce shRNA expression. Steady-state levels of mRNAs encoding Drosha and several primary miRNA transcripts were measured by qRT-PCR, and normalized to levels of the *RpL32* mRNA. RNA isolated from *da>gfp shRNA* males serves as negative control. (n = 3). (**B**) Flies were left untreated (- *E*. *coli*) or infected by *E*. *coli* via septic injury (+ *E*. *coli*), total RNAs were extracted 6 hours post-infection and mRNAs encoding the AMP Diptericin was measured and normalized to levels of *RpL32* (n = 5; mean + standard deviation (**SD**)). (**C-E**) Select miRNAs were over-expressed in flies by crossing UAS-miRNA transgenic lines *da>Gal4 tub-Gal80* ^*ts*^ flies. Flies crosses were kept at 18°C and progeny were shifted to 29°C for 5 days upon eclosure to induce miRNA expression. (**C**) Northern blot shows levels of select miRNAs (right) in control and miRNA over-expression flies. 2S rRNA serves as loading control. In addition, flies were either uninfected (**D**) or infected with *E*. *coli* via septic injury (**E**). Total RNA was isolated from flies 6 hrs post-infection and levels of *Diptericin* mRNA were measured by RT-qPCR and normalized to the *RpL32* mRNA. RNA samples from *da>gfp shRNA* flies serves as control. Note that levels of the *Diptericin* mRNA in non-infected and *E*. *coli*-infected *da>gfp shRNA* flies serve as baseline controls in both **D** and **E** (n≥4). (**F**) A Northern blot shows *miR-34* expression levels in naïve S2 cells and *miR-34* overexpression cells (both were treated with 20-HE at 1 μM for 24 hrs). (**G**) S2 cells over-expressing *miR-34* and control cells were both treated with 20 hydroxy-ecdysone (**20-HE**) at 1 μM for 24 hrs. Subsequently cells were either left untreated or treated for 6 hrs with a crude lipopolysaccharide sample at 10 μg/mL, which contains the immune stimulator *p*eptido*g*lyca*n* (**PGN**). Total RNA was isolated and levels of *Diptericin* mRNA were measured by RT-qPCR and normalized to the *RpL32* mRNA (n = 3). (**H**) Canonical components of IMD signaling were depleted in *miR-34* over-expressing cells using dsRNAs targeting IMD pathway components (below) or a control dsRNA against the firefly luciferase gene. Cells were first treated with 20-HE for 24 hours, and subsequently were either left untreated or treated with PGN, and levels of *Diptericin* mRNA were measured by RT-qPCR and normalized to the *RpL32* mRNA (n = 3). (**I**) *UAS-miR-34* or the control *UAS-sh-gfp* flies were crossed to *da>Gal4 tub-Gal80* ^*ts*^ flies. Flies crosses were kept at 18°C. Upon eclosure, progeny of appropriate genotypes were collected and shifted to 29°C for 7 days. Flies in groups of 45 were subsequently injected with a concentrated culture of *Erwinia carotovora carotovora 15* (*Ecc15*) or PBS (non-infection control) and kept at 29°C. Fly survival was recorded daily up to day 8 post-infection and plotted (n≥3; *p*<0.05 between *Ecc15*-infected control and *miR-34* ^*OX*^ (*da>miR-34 Gal80* ^*ts*^) flies). (**J-K**) Control or *miR-34* ^*OX*^ flies were infected by injecting a concentrated culture of *Ecc15* (**J**) or overnight culture of *Enterobacter cloacae* (**K**). At various time points post-infection, groups of 3 flies in **J** (and groups of 4 flies in **K**) were collected and homogenized in sterile PBS. Fly homogenates were diluted and plated onto Ampicillin- (in **J**) or Nalidixic acid-containing (in **K**) LB plates, and the resultant colonies were counted one day later. Shown are *c*olony-forming *u*nits (**CFU**s) per fly (n≥4).

The ribonuclease Drosha is a core component of the miRNA processing machinery essential for the biogenesis of most miRNAs [60]. To examine whether defects in miRNA biogenesis affects innate immunity signaling, we first silenced *Drosha* expression *in vivo* by crossing a shRNA transgenic line targeting *Drosha* (***sh-Drosha***) to the *daughterless-Gal4* (***da-Gal4***)*; tub-Gal80* ^*ts*^ composite line [61]. A control cross was set up between a shRNA transgenic line targeting *gfp* and *da-Gal4; tub-Gal80* ^*ts*^ flies. To minimize lethality due to the requirement for select miRNAs in development, fly crosses were kept at permissive temperature (18°C). Upon eclosure progeny of appropriate genotype was shifted to restrictive temperature (29°C) for 5 days to allow for shRNA transgene expression and target gene silencing. This strategy allowed us to achieve significant knockdown of the *Drosha* mRNA, as measured by RT-qPCR (Fig 1A). Consistent with the critical requirement for Drosha in miRNA biogenesis, depletion of Drosha leads a marked accumulation of several primary miRNA transcripts compared with a control knockdown (*tub-Gal80* ^*ts*^ *da>sh-gfp*) (Fig 1A). Importantly, we found that *tub-Gal80* ^*ts*^ *da>sh-Drosha* flies display a decrease in AMP expression levels compared with control animals, under both non-infection and *E*. *coli* infection conditions (Fig 1B). Interestingly, crosses between *da-Gal4* and *shRNA-Drosha* at room temperature yielded under-represented but viable *da>sh-Drosha* progeny. As expected, we detected a decrease in levels of the *Drosha* mRNA and an accumulation of primary miRNA transcripts in these flies (S1 Fig). However, in contrast to the observed decrease in AMP expression in *tub-Gal80* ^*ts*^ *da>sh-Drosha* flies, *da>sh-Drosha* flies display elevated basal levels of AMP expression compared with control animals (S1B Fig). The seemingly disparate phenotype in AMP expression in *da>sh-Drosha* progeny between the presence and absence of *tub-Gal80* ^*ts*^ could be attributable to the difference in the onset and duration of Drosha depletion: While Drosha depletion occurred in *da>sh-Drosha* animals throughout development, it did not take place in *tub-Gal80* ^*ts*^ *da>sh-Drosha* flies until fully developed adult animals were shifted to the restricted temperature. For instance, the barrier function of the digestive tract could be compromised in *da>sh-Drosha* animals due to a requirement for Drosha/miRNAs during development, leading to escape of the microbes and an increase in AMP expression. Nonetheless, these data demonstrate that dysregulation of miRNA biogenesis as a whole impacts the IMD innate immunity signaling pathway.

### Over-expression of *miR-34* leads to hyperactivation of innate immunity signaling both in cultured cells and *in vivo*

To further reveal the identities of cellular miRNAs that underlie the innate immunity phenotype, we conducted a miRNA over-expression screen *in vivo* using 101 UAS-miRNA transgenic lines and the *da-Gal4; tub-Gal80* ^*ts*^ composite line [62]. A control cross was set up between a shRNA transgenic line, which expresses an artificial sh-*gfp* RNA embedded in the *miR-1* cassette [61], and *da-Gal4; tub-Gal80* ^*ts*^ flies. Fly crosses were kept at permissive temperature (18°C) until adult progeny of the appropriate genotype emerged. Subsequently the progeny was shifted to restrictive temperature (29°C) for 5 days to allow for miRNA transgene expression. This strategy allowed us to achieve marked over-expression of individual miRNAs. Fig 1C shows results of Northern blot analyses for select miRNAs. Under these experimental conditions, viable adult progeny of the appropriate genotype that over-expresses individual miRNAs was readily recovered. One exception was *let-7*, which causes lethality at pupal stage (S1 Table). Subsequently, these flies were either left untreated or infected with *E*. *coli* via septic injury. Total RNA was isolated 6 hours post-infection and levels of the *Diptericin* (***Dpt***) mRNA were measured and normalized to the *RpL32* control mRNA. S1 Table documents the relative *Diptericin* mRNA levels in uninfected and *E*. *coli*-infected flies compared to the respective controls (*da>sh-gfp* flies that are uninfected and infected by *E*. *coli*). The AMP expression phenotype for select miRNAs (*miR-34*, *miR-92a*, *miR-9a* and *miR-989*) is shown (Fig 1D and 1E). In subsequent studies, we focused on *miR-34* because 1) *miR-34* over-expression causes a concordant increase in levels the *Diptericin* mRNA in both uninfected and *E*. *coli*-infected flies; and 2) *miR-34* expression is regulated by ecdysone signaling, which impacts innate immunity [33, 63].

Besides IMD signaling, which is activated upon Gram-negative bacterial infection, we also asked whether *miR-34* additionally impacts other signaling routes, such as the Toll signaling pathway, which mediates host defense against infection by fungi and Gram-positive bacteria. Interestingly, our analysis revealed that *Drosomycin* expression in response to *M*. *luteus* infection was significantly decreased in *miR-34* over-expressing flies compared with controls (S2 Fig), suggesting that *miR-34* differentially impacts IMD and Toll signaling. Next, we examined the impact of *miR-34* over-expression on AMP expression in immuno-competent cultured S2 cells. We detected higher levels of the *Diptericin* mRNA, as well as a panel of mRNAs encoding additional AMPs, including *Cecropin A1* (*CecA1*), *Attacin A* (*AttA*), *Metchnikowin* (*Mtk*) and *Defensin* (*Def*), in *miR-34* overexpressing cells than in control cells, both in the absence and presence of peptidoglycan (PGN) treatment (Fig 1F and 1G, S3 Fig). In addition, levels of the *pirk* mRNA, which is transcriptionally activated by IMD signaling and encodes a negative regulator of IMD signaling, were also increased both in untreated and PGN-treated cells upon *miR-34* over-expression (S4 Fig). Importantly, depletion of core components of the IMD signaling pathway, such as PGRP-LC, subunits of the DmIKK complex (Ird5 and Kenny), or Relish, significantly alleviated the immune-activation phenotype of *miR-34* (Fig 1H), indicating that the canonical IMD signaling pathway is required for the immune-stimulating function of *miR-34*. Interestingly, in ecdysone-treated S2 cells the effect of *miR-34* overexpression appears to be PGRP-LC-dependent, even in the absence of PGN stimulation. It is possible that *miR-34* overexpression may impact ecdysone-mediated regulation of *PGRP-LC* expression [33], thereby affecting IMD signaling. Taken together, these data demonstrate that mis-expression of *miR-34* affects the IMD innate immunity signaling pathway both in cultured cells and *in vivo*.

### *miR-34* impacts innate immunity signaling *in vivo*

To further define the role of *miR-34* in innate immunity signaling *in vivo*, we injected either sterile PBS or a suspension of a concentrated culture of the fly pathogen *Erwinia carotovora carotovora* strain 15 (*Ecc15*) into *miR-34* over-expression or control flies, and monitored fly survival at different time points post injection. As expected, flies of both genotypes survive well in response to PBS injection (Fig 1I). Importantly, while flies of both genotypes show a decrease in survival following *Ecc15* infection, *miR-34* over-expression flies display a significantly higher survival rate than control flies (Fig 1I), consistent with the observed higher levels of AMP expression in *miR-34* over-expression flies. To further examine whether enhanced survival of *miR-34* over-expression flies upon *Ecc15* infection is attributable to improved pathogen clearance or immune tolerance, we monitored pathogen load at various time points post *Ecc15* infection. Our analysis revealed that while infected flies of both genotypes carry comparable amount of *Ecc15* at the starting time point, *miR-34* over-expression flies appear to out-perform control animals in clearing invading pathogens, as indicated by the lower pathogen load in *miR-34* over-expression flies than in controls (Fig 1J). Similar observations were made upon infecting flies with, *Enterobacter cloacae*, a second Gram-negative bacteria (Fig 1K). Furthermore, to examine whether *miR-34* over-expression affects hemocyte-mediated bacterial phagocytosis, which is a key mechanism of the cellular immune response, we injected into flies *E*. *coli* bio-particles conjugated with a PH-sensitive dye, which becomes fluorescent only after being engulfed by hemocytes and sorted into the acidic endosomal compartment. This analysis reveals a similar degree of phagocytosis between control and *miR-34* over-expression flies (S5A and S5B Fig). We conclude that *miR-34* over-expression enhances innate immunity and host survival upon Gram-negative bacterial infection, at least in part, by promoting AMP expression and pathogen clearance.

**Fig 2:**
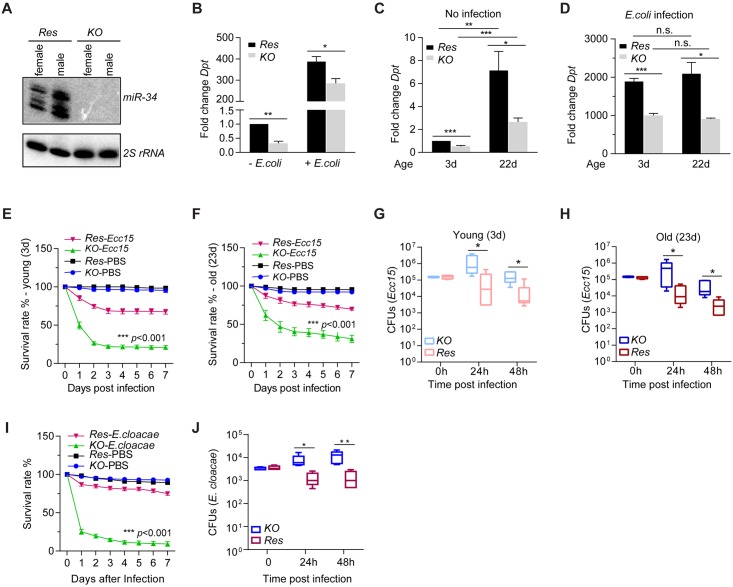
*miR-34* deficiency compromises innate immunity. (**A**) A Northern blot shows levels of *miR-34* and the control *2S* rRNA in *miR-34* knockout flies (***KO***) or knockout flies carrying a *miR-34* rescue transgene (control, ctr). (**B**) Flies were either uninfected or infected with *E*. *coli* via septic injury. Total RNA was isolated and levels of the *Diptericin* mRNA were measured by RT-qPCR and normalized to the *RpL32* control (mean + SD; n = 3). (**C-D**) *miR-34* ^*KO*^ and control flies were reared in standard food supplemented with antibiotics. Age-matched fly progeny (young– 3d, old– 22d) were either uninfected (**C**) or infected with *E*. *coli* via septic injury (**D**), and levels of the *Diptericin* mRNA were measured by RT-qPCR and normalized to the *RpL32* control (n≥3). In both panels, the *Dpt*/*RpL32* ratio in non-infected 3d old control flies serves as baseline. (**E-F**) Groups of 45 age-matched *miR-34* ^*KO*^ and control flies (**E**, young– 3d; **F**, old– 23d) were injected with a concentrated culture of *Ecc15* or PBS. Fly survival was recorded daily and plotted (n≥3; *p*<0.001 between *Ecc15*-infected control and *miR-34* ^*KO*^ flies). (**G-H**) A similar group of age-matched control or *miR-34* ^*KO*^ flies (as in **E** and **F**) were infected by injecting a concentrated culture of *Ecc15*. At various time points post-infection, groups of 3 flies were collected and homogenized in sterile PBS. Note that due to lethality, 1 fly per group was used for a subset of data points in *miR-34* ^*KO*^ flies 2 days post-infection. Fly homogenates were diluted and plated onto Ampicillin-containing LB plates, and the resultant colonies were counted one day later. Shown are *c*olony-forming *u*nits (**CFU**s) per fly (n≥5). (**I**) Groups of 4–7 d *miR-34* ^*KO*^ and control flies were injected with a concentrated culture of *Enterobacter cloacae* or PBS. Fly survival was recorded daily and plotted (n≥3; *p*<0.001 between *Enterobacter cloacae*-infected control and *miR-34* ^*KO*^ flies). (**J**) A similar set of flies (as in **I**) were infected by injecting an overnight culture of *Enterobacter cloacae*. At various time points post-infection, groups of 4 flies were collected and homogenized in sterile PBS. Fly homogenates were diluted and plated onto Nalidixic acid-containing LB plates, and the resultant colonies were counted one day later. Shown are *c*olony-forming *u*nits (**CFU**s) per fly (n = 5).

Next, we examined whether *miR-34* deficiency impacts innate immunity by measuring AMP expression both in *miR-34* knockout (***miR-34*** ^***KO***^) flies [64] and in control (***Res***) animals (*miR-34* ^*KO*^ flies carrying a *miR-34* genomic rescue construct) (Fig 2A). This analysis revealed that *miR-34* ^*KO*^ flies express significantly lower levels of the *Diptericin* mRNA than control animals, under both non-infection and *E*. *coli* infection conditions (Fig 2B). To alleviate the concern that endogenous or environmental microbes could potentially impact host innate immunity and cause variations in levels of AMP expression, we also analyzed *miR-34* ^*KO*^ and wildtype flies raised in media containing multiple antibiotics. We note that antibiotics treatment alone may not completely eliminate microbes. Nonetheless, under these conditions, lower levels of *Diptericin* expression was detected in *miR-34* ^*KO*^ animals than in control animals (Fig 2C and 2D). Consistent with lower levels of *Dpt* expression, *miR-34* ^*KO*^ flies present poorer survival and pathogen clearance in response to *Ecc15* compared to control animals (Fig 2E). Lastly, since *miR-34* expression steadily increases with age, we also examined AMP expression, host survival and pathogen clearance upon *Ecc15* infection in various groups of age-matched *miR-34* ^*KO*^ and control flies. Consistent with previous reports [65–67], we detected higher basal levels of *Diptericin* transcript in aged control flies than in young flies (Fig 2C). Notably, this age-dependent increase in basal levels of AMP expression persists in *miR-34* ^*KO*^ animals, suggesting that age-dependent increase in *miR-34* expression does not significantly contribute to the observed difference in AMP expression between young and old flies. Importantly, in both young and aging settings, *miR-34* ^*KO*^ flies display lower basal and *E*. *coli* infection-induced levels of *Diptericin* expression, poorer survival and pathogen clearance in response to *Ecc15* challenge compared to age-matched control animals (Fig 2C–2H). Lastly, our analysis reveals that *miR-34* ^*KO*^ flies display a defective survival and pathogen clearance in response to *Enterobacter cloacae* infection (Fig 2I and 2J), and that *miR-34* deficiency did not significantly impact hemocyte-mediated phagocytosis (S5C and S5D Fig). These data demonstrate that *miR-34* deficiency compromises AMP expression and impairs IMD signaling.

### Identification of *miR-34* target genes relevant to innate immunity signaling

**Fig 3:**
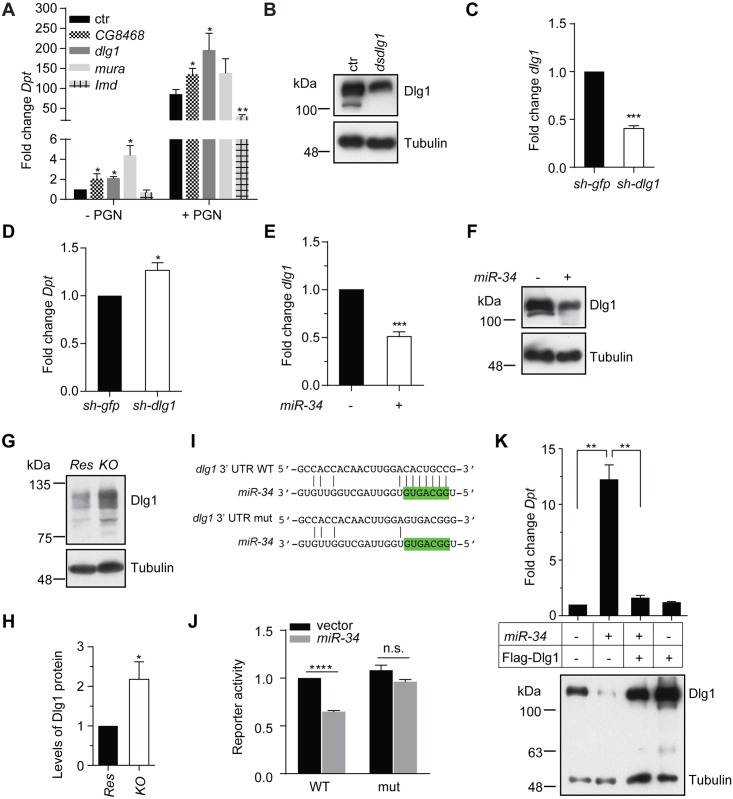
*dlg1* is a *miR-34* target gene relevant to innate immunity signaling. (**A**) S2 cells treated with various dsRNAs (below) were first treated with 20-HE for 24 hrs, and were subsequently either left untreated or treated with PGN. Total RNA was isolated and levels of *Diptericin* were measured and normalized to *RpL32* (n = 3). *Imd* dsRNA serves as a positive control. (**B**) Levels of Dlg1 protein in *dlg1* knockdown cells or control cells were measured by immunoblot. Tubulin serves as a loading control. The remaining Dlg1 protein reflects incomplete depletion. (**C-D**) *Dlg1* was knocked down in flies using a UAS-shRNA and the *da-Gal4 tub-Gal80* ^*ts*^ transgenes in a similar strategy as described in Fig 1. Levels of *dlg1* (**C**) and *Diptericin* mRNA (**D**) in *E*. *coli*-infected flies were measured by qRT-PCR, and normalized to *RpL32*. RNA isolated from progeny of a parallel cross using the *sh-gfp* transgene serves as negative control (n = 3). (**E-F**) Levels of *dlg1* mRNA (**E**) or protein (**F**) in S2 cells over-expressing *miR-34* or in control cells were measured. The *RpL32* mRNA and Tubulin protein serve as controls, respectively (n = 3). The remaining Dlg1 protein in **F** reflects incomplete depletion. (**G-H**) Levels of Dlg1 protein in *miR-34* knockout or control flies were measured by immunoblot (**G**). Note that multiple Dlg1 protein isoforms were detected in fly extracts. Their collective abundance was quantified and shown in **H** (n = 3; mean + SD). (**I**) *Renilla* luciferase reporter constructs that carry the *dlg1* 3’ UTR containing either a wildtype (**WT**) or mutant (**mut**) *miR-34* binding site were generated. Seed region of *miR-34* was highlighted in green. (**J**) The *Renilla* luciferase reporter constructs (in **J**) together with a control firefly luciferase construct were transfected into S2 cells with or without a *miR-34* expression vector. Reporter activities were measured and relative *Renilla*/Firefly ratio is shown (n = 3). (**K**) Various combinations of *miR-34* and Flag-Dlg1 expression constructs were transfected into S2 cells. Total RNA was isolated and levels of the *Diptericin* mRNA were measured and normalized to *RpL32* (upper panel; n = 3). Levels of Dlg1 protein were measured by immunoblot (lower panel).

To identify *miR-34* targets, we first employed two widely used bioinformatics algorithms (TargetScan and PicTar) [46, 68, 69]. In addition, we performed mRNA sequencing and compared the mRNA expression profiles in cells over-expressing *miR-34* with that in control samples. As most miRNAs only mildly reduce the expression of cognate target genes, we undertook an inclusive approach and considered all mRNAs that display a decrease in expression upon *miR-34* over-expression. Integration of these datasets identified a list of 27 genes that not only scored positively using both bioinformatics algorithms, but also displayed a decrease in mRNA levels upon *miR-34* over-expression (S6 Fig & S2 Table). Among these 27 genes is *Eip74EF*, which is a well-characterized *miR-34* target, thereby validating our approach [64]. Next, to examine whether any of these 27 genes are functionally relevant to innate immunity signaling, we knocked down these genes individually in S2 cells and examined the impact on *Diptericin* expression prior to and after PGN treatment. This analysis revealed that inactivation of three genes, *CG8468*, *dlg1* and *mura*, led to an increase both in basal levels and/or PGN-induced *Diptericin* expression (Fig 3A). While the magnitude of changes in *Diptericin* expression upon silencing of individual genes appears to be weaker than that elicited by *miR-34* overexpression, possibly due to low knockdown efficiency and/or slow turnover of target proteins, these data nonetheless suggest that *CG8468*, *dlg1* and *mura* are candidate *miR-34* target genes that could potentially downregulate IMD signaling.

### Validating *dlg1* and *Eip75B* as *bona fide miR-34* target genes that modulate innate immunity signaling

Dlg1 is a member of *m*embrane-*a*ssociated *gu*anylate *k*inase (**MAGUK**) family proteins (albeit it lacks catalytic activity), and is localized to septate junctions [70]. Depletion of Dlg1 in cultured S2 cells led to an increase in both basal and PGN-induced *Diptericin* expression (Fig 3A and 3B). In addition, we assessed the impact of Dlg1 depletion on innate immunity signaling in flies using UAS-shRNA transgenic lines (*sh-dlg1* and the *sh-gfp* control) and the *da-Gal4; tub-Gal80* ^*ts*^ composite line. As expected, levels of the *dlg1* mRNA are reduced in *da>sh-dlg1* flies compared with that detected in the control *da>sh-gfp* flies (Fig 3C). Importantly, such a decrease in *dlg1* mRNA levels correlated with an increase in levels of the *Diptericin* mRNA (Fig 3D). These data demonstrate that a reduction in levels of Dlg1 correlates with enhanced IMD signaling both in cultured cells and *in vivo*. Consistent with the notion that *dlg1* is a *miR-34* target gene, we observed a reduction in both mRNA and protein levels of *dlg1* upon over-expression of *miR-34* (Fig 3E and 3F). In addition, higher levels of Dlg1 protein were detected in *miR-34 k*nock*o*ut flies than in control animals (Fig 3G and 3H). Furthermore, our bioinformatics analysis predicted a *miR-34* binding site in the *dlg1 3’ u*n*t*ranslated *r*egion (**3’ UTR**) (Fig 3I). To show definitively that the predicted *miR-34* binding site is sufficient to confer gene silencing, we performed reporter assays using luciferase reporter constructs carrying either the wildtype or mutant *dlg1* 3’ UTR, which abolishes *miR-34* binding (Fig 3I). This analysis reveals that *miR-34* is capable of silencing the reporter construct carrying a wildtype *dlg1* 3’ UTR, and this repression is relieved by introducing mutations in the *miR-34* binding site (Fig 3J). Lastly, we found that while *miR-34* over-expression led to a significant reduction in levels of Dlg1 protein, expression of a *dlg1* cDNA construct containing only the open reading frame efficiently restored Dlg1 protein levels in S2 cells even in the presence of exogenous *miR-34* (Fig 3K, lower panel). This is consistent with the notion that a functional *miR-34* target site is present only in the 3’ UTR of the *Dlg1* mRNA. Importantly, expression of *dlg1* efficiently blunted the immune-stimulating effect of *miR-34* (Fig 3K, upper panel). Taken together, these data demonstrate that *dlg1* is a *bona fide* (perhaps a major) *miR-34* target gene relevant to IMD signaling.

**Fig 4:**
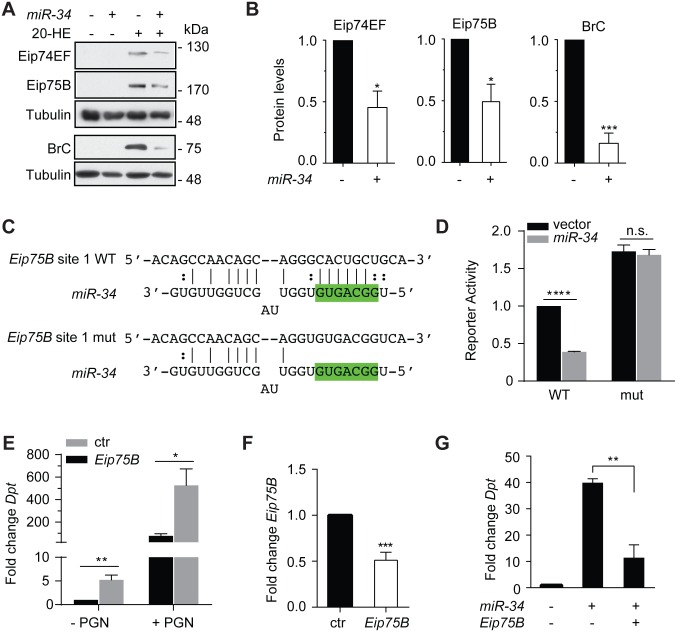
*Eip75B* is another *miR-34* target gene that modulates innate immunity signaling. (**A-B**) S2 cells over-expressing *miR-34* or control cells were either left untreated or treated with 20-HE (labeled on top). Cell lysates were subject to immunoblot using various antibodies against Eip74EF, Eip75B, BrC or the control Tubulin (**A**). Levels of the indicated proteins in 20-HE-treated cell samples were quantified in **B** (n≥3). (**C**) Reporter constructs were generated that carry either a wildtype (WT) or mutant (mut) *miR-34* binding site derived from the *Eip75B* ORF. Seed region of *miR-34* was highlighted in green. (**D**) The reporter constructs were transfected into S2 cells together with or without a *miR-34* expression construct, and reporter activities were measured (n = 3). (**E-F**) S2 cells treated with dsRNA against *Eip75B* or a control dsRNA were first treated with 20-HE, and subsequently were either left untreated or treated with PGN. Total RNA was isolated and levels of the *Diptericin* (**E**) and *Eip75B* mRNA (**F**) were measured and normalized to *RpL32* (n≥3). (**G**) Various combinations of *miR-34* and *Eip75B* expression constructs were transfected into S2 cells. Cells were treated with 20-HE for 24 hrs, and total RNA was isolated and levels of the *Diptericin* mRNA were measured and normalized to the *RpL32* control mRNA (n = 3).

Among our list of *miR-34* targets is *Eip74EF*, which encodes a key component of the ecdysone signaling cascade and has been previously identified as a *miR-34* target gene [64]. We confirmed these findings by showing that over-expression of *miR-34* can lead to a significant reduction in the levels of Eip74EF protein (Fig 4A and 4B). Inspired by the notion that a single miRNA can coordinately regulate the expression of multiple components of a given pathway [71], we performed immunoblot assay and surveyed additional ecdysone response genes to search for additional *miR-34* targets. This analysis revealed that protein levels of Eip75B, a transcriptional factor and component of ecdysone signaling, are significantly reduced by *miR-34* over-expression (Fig 4A and 4B). Further inspection of the *Eip75B* mRNA sequence allowed us to identify five potential *miR-34* sites in the coding region. In order to test whether any of these sites can confer *miR-34*-mediated gene silencing, we generated reporter constructs by placing tandem triple repeats of individual *miR-34* binding sites derived from the *Eip75B* mRNA in the 3’ UTR of the firefly luciferase gene, and examined whether these reporter genes can be repressed by *miR-34*. In addition, we generated a corresponding set of reporter constructs carrying mutations in candidate *miR-34* binding sites that abolish *miR-34* recognition. This analysis reveals that reporter genes containing wild type sites #1 or #5 were more efficiently repressed by *miR-34* than the reporter with mutant sites (Fig 4C and 4D & S7 Fig). In addition, depletion of Eip75B in S2 cells resembles the *miR-34* over-expression phenotype, i.e. activation of innate immunity signaling both in the presence and absence of PGN treatment (Fig 4E and 4F). These observations are consistent with previous studies that identified Eip75B as a negative regulator of IMD signaling [33, 72]. Importantly, over-expression of the *Eip75B-RC* isoform, which lacks site #1, significantly suppressed the immune-activation function of *miR-34* (Fig 4G). We conclude that besides *dlg1*, *Eip75B* is another *miR-34* target gene relevant to innate immunity signaling.

### *miR-34* is transcriptionally repressed by ecdysone signaling

**Fig 5:**
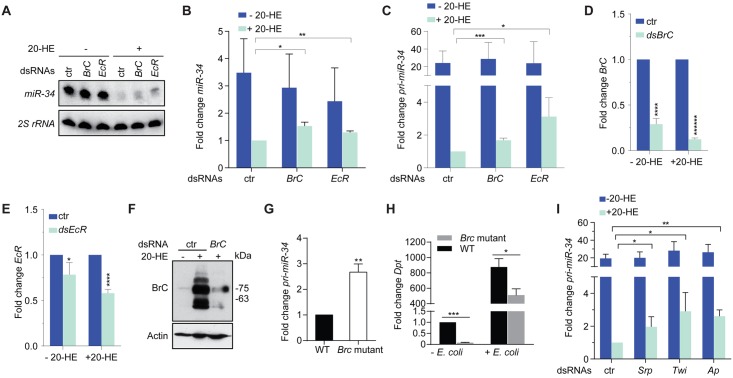
Identification of trans-acting transcription factors required for ecdysone-mediated repression of *miR-34*. (**A-F**) S2 cells transfected with various dsRNAs were left untreated or treated with ecdysone (20-HE) at 1 μM for 48 hrs. Total RNA was isolated and levels of mature *miR-34* were measured by Northern blot (**A**) and normalized to the 2S rRNA (**B**; n = 3). In addition, levels of the primary *miR-34* transcript (**C**), or the mRNAs for *BrC* (**D**) or *EcR* (**E**) were measured and normalized to the control *RpL32* mRNA (n = 3; mean + SD). In addition, levels of the BrC protein were measured by immunoblot (**F**). Note that multiple isoforms of the BrC protein are expressed in S2 cells upon ecdysone treatment and are responsive to dsRNA-mediated knockdown. (**G**) RNA was extracted from third instar *BrC* ^*npr6*^ mutant or wildtype (WT) larvae and levels of *pri-miR-34* were measured and normalized to the control *RpL32* mRNA (n = 4). (**H**) *BrC* ^*npr6*^ mutant and wildtype larvae was either left untreated or infected by a concentrated culture of *E*. *coli* via septic injury. Total RNA was isolated 6 hrs post-infection and levels of *Dpt* mRNA were measured and normalized to the control *RpL32* mRNA (n = 4). (**I**) S2 cells transfected with dsRNAs targeting various transcription factors (below). These cells were left untreated or treated with ecdysone, and levels of *pri-miR-34* were measured and normalized to the control *RpL32* mRNA (n≥3; mean + SD).

It has been reported that *20*-*h*ydroxy*e*cdysone (**20-HE**) treatment in S2 cells can profoundly alter the expression levels of a number of miRNAs. For example, expression of *let-7*, *miR-100* and *miR-125*, which cluster at the same genomic locus, is markedly induced upon 20-HE treatment [63]. In contrast, levels of *miR-34* display a significant decrease in response to 20-HE treatment. We confirmed the inhibitory effect of 20-HE on *miR-34* expression by performing Northern blot to quantify levels of mature *miR-34* in S2 cells before and after 20-HE treatment (Fig 5A and 5B). Such ecdysone-mediated modulation of *miR-34* gene expression is most likely at the level of transcription, as changes in levels of the primary miRNA transcripts resemble that of mature miRNAs (Fig 5A–5C) [63]. Further supporting this notion, a *miR-34* transgene driven by the *metallothionein* promoter was robustly expressed in the presence of 20-HE, while its endogenous counterpart under the control of cognate regulatory elements was strongly repressed under the same conditions (Fig 1F).

*Ec*dysone *r*eceptor (**EcR**) and the *Br*oad *c*omplex (**BrC**) are essential for ecdysone signaling. EcR heterodimerizes with its co-factor Ultraspiracle, and functions as a stereotypical steroid hormone receptor to modulate the expression of ecdysone target genes, whereas the transcription factor BrC is an ecdysone-induced early gene product that regulates the expression of downstream ecdysone-responsive genes [29, 73, 74]. Consistent with previous reports showing that *let-7* expression is activated by ecdysone signaling, depletion of ecdysone EcR or BrC in S2 impaired the increase in levels of the *pri-let-7* transcript after 20-HE treatment (Fig 5D and 5E & S8 Fig) [59, 63]. In addition, while BrC has been implicated in ecdysone-mediated repression of *miR-34*, the requirement for BrC in this process could not be definitively assessed [63]. We therefore measured changes in levels of both the primary *miR-34* transcript and mature *miR-34* elicited by *BrC* or *EcR* knockdown. This analysis revealed that the repressive effect of ecdysone on *miR-34* expression was partially relieved in *EcR* or *BrC* knockdown cells compared with control RNAi cells (Fig 5A–5F). Furthermore, consistent with the findings of Silverman, Ambros and colleagues [33, 63], we detected higher levels of *pri-miR-34* in *BrC* mutant larvae than in control animals, and found that the *BrC* mutant larvae expressed lower levels of *Dpt* in response to *E*. *coli* infection (Fig 5G and 5H). Taken together, these data demonstrate that both EcR and BrC are required for optimal ecdysone-mediated transcriptional repression of *miR-34*.

We noticed that while *BrC* dsRNA treatment led to a significant reduction in both mRNA and protein levels of BrC, 20-HE-mediated repression of *miR-34* was only partially relieved (Fig 5A–5F). These observations suggest that there are yet-to-be-identified factors that are required for 20-HE-mediated *miR-34* repression. A recent study reported that the consensus binding motif for a collection of transcriptional factors, including Pnr, Aef1, Trl, CrebA, Srp, Twi and Ap, are enriched in candidate regulatory regions that confer ecdysone-mediated transcriptional repression [75]. We therefore examined whether these transcription factors contribute to ecdysone-mediated repression of *miR-34*. In addition, we also included in our test additional components of the ecdysone signaling pathway, such as Hr46, E78C and E93F. Our analysis revealed that three additional transcription factors (Srp, Twi and Ap) are required for optimal repression of *miR-34* by ecdysone (Fig 5I, S9 Fig).

### Identification of *cis*-regulatory elements responsible for ecdysone-mediated repression of *miR-34*

**Fig 6:**
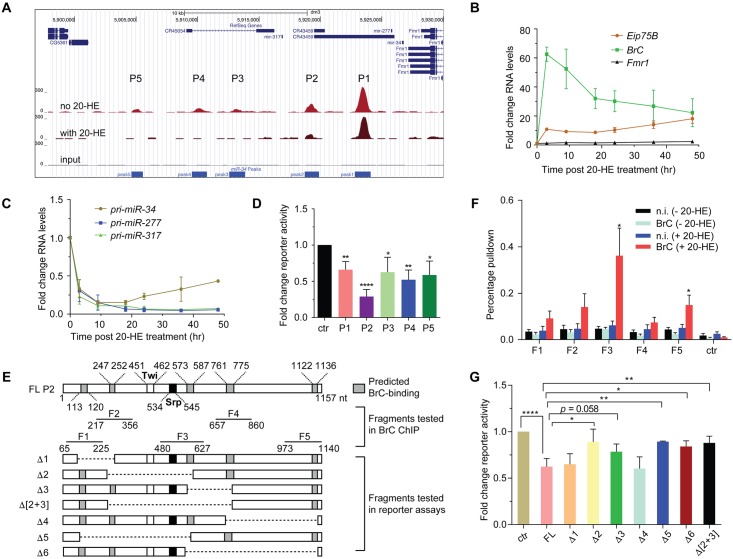
Mapping *cis*-regulatory elements required for ecdysone-mediated repression of *miR-34*. (**A**) The *miR-34* locus contains five candidate regulatory regions (***P1* through *P5***) that are repressed in response to ecdysone treatment. (**B-C**) S2 cells were treated with ecdysone for various times (below) and levels of various mRNAs (**B**) or pri-miRNAs (**C**) were measured and normalized to the control *RpL32* mRNA (n≥3). (**D**) DNA fragments derived from various regulatory regions were placed upstream of a luciferase reporter gene. S2 cells transfected with these reporter constructs were left untreated or treated with ecdysone (20-HE) and reporter activities were measured. Fold change in ecdysone-mediated repression of reporter activity is shown (n≥3; mean + SD). Cells transfected with a firefly luciferase reporter gene driven by the regulatory region derived from the *traffic jam* gene, which is not responsive to ecdysone treatment, serve as control. (**E**) A schematic of the *P2* region. Predicted BrC-binding sites are shown as shaded boxes, whereas open and filled boxes represent one of the three predicted Twi- and Srp-binding sites, respectively. F1-F5 represent various fragments tested for BrC occupancy in **D**. In addition, various truncated fragments tested in reporter assays in **G** are shown on the left. (**F**) Chromatin immunoprecipitation assay was employed in cultured S2 cells to measure BrC occupancy in various regions of *P2* (schematic shown in **E**, n = 3; mean + SD). (**G**) Reporter constructs containing either full length or various truncated *P2* fragments (in **E**) were transfected into S2 cells. Cells were left untreated or treated with ecdysone (20-HE) and reporter activities were measured. Ecdysone-mediated repression of reporter activity is shown (n≥3; mean + SD).

The *miR-34* genomic locus contains two additional miRNA genes, *miR-277* and *miR-317*, as well as a protein-coding gene *Fmr1* (Fig 6A). While all the three miRNA genes are repressed by 20-HE treatment, little changes in levels of the *Fmr1* mRNA were detected (Fig 6B and 6C). As a control, 20-HE treatment led to a marked increase in levels of the *BrC* and *Eip75B* mRNA (Fig 6B). These observations suggest that local regulatory sequences may confer 20-HE-mediated repression of these miRNA genes. Recently the Stark group has employed STARR-seq (self-transcribing active regulatory region sequencing) to identify enhancers at a genome-scale [76]. STARR-seq exploits the ability of enhancers to function independently of their relative positions. If candidate enhancers are placed downstream of a minimal promoter, such that active enhancers transcribe themselves, each enhancer's strength will be reflected by its abundance among cellular RNAs. This approach has identified several genomic regions near the *miR-34* locus (referred to as ecdysone-responsive peaks P1 through P5) as putative ecdysone-responsive elements (Fig 6A) [75]. To examine whether these DNA elements can mediate ecdysone-dependent repression, we generated reporter constructs by placing individual genomic fragments upstream of a *Drosophila* Synthetic Core Promoter (**DSCP**) that drives the firefly luciferase reporter gene. Reporter assays reveal that each of the five regions can confer ecdysone-mediated repression with varying strengths. Notably, among the five genomic fragments tested, peak P2 displays the most robust repressive effect in response to ecdysone treatment (Fig 6D). Of note, all three ecdysone-repressed miRNA genes are transcribed in the same direction, whereas the ecdysone-insensitive *Fmr1* gene is transcribed in the opposite direction to the miRNA genes. Consistent with the notion that peak P2 is the major regulatory element that confers sensitivity to ecdysone signaling, it is physically located within a closer distance to the miRNA genes than to the *Fmr1* gene (Fig 6A).

Since BrC is required for optimal ecdysone-mediated repression of *miR-34*, we inspected the nucleotide sequence of peak P2 and identified five putative BrC-binding sites (shown as shaded boxes in Fig 6E). To validate and assess BrC occupancy on these regions *in vivo*, we performed *ch*romatin *i*mmuno*p*recipitation (**ChIP**) assay both in naïve S2 cells and cells treated with 20-HE, using an antibody against BrC. As expected, in the absence of ecdysone treatment, BrC is expressed at very low levels and thus no enrichment can be detected. However, upon 20-HE treatment, we detected various degree of BrC enrichment in these regions, with F3 displaying the most significant BrC occupancy compared with a control ChIP using a *n*on-*i*mmune (**n.i.**) serum (Fig 6F).

To further define the minimal *cis*-regulatory element within peak P2 that are required for *miR-34* repression, we placed various truncated P2 genomic fragment upstream of the reporter gene and assessed their capability of conferring ecdysone-mediated silencing. This analysis revealed that any deletions that perturb the region composed of nucleotides 573–587 compromised the repressive effect of ecdysone (Fig 6E and 6G). Notably, the genomic fragment F3, which displays the highest degree of BrC occupancy, centers around nucleotides 573–587 (Fig 6E and 6F), suggesting that BrC binding at this region is required, at least in part, for the repressive effect of ecdysone on *miR-34* expression. Taken together, these analyses identified key *cis*-regulatory genomic elements and trans-acting transcription factors required for ecdysone-mediated repression of *miR-34*.

## Discussion

In this study, we identify the microRNA *miR-34* as a link in the intricate interplay between ecdysone signaling and innate immunity. We show that over-expression of *miR-34* either in flies or in cultured S2 cells leads to hyper-activation of antimicrobial peptide gene expression both in the absence and in the presence of immune challenge, and enhances pathogen clearance *in vivo*, and that *miR-34* deficiency compromises innate immunity. In addition, *miR-34* modulates IMD signaling, in part, by repressing genes encoding the septate junction protein Dlg1 as well as Eip75B, a component of the ecdysone signaling cascade and a negative regulator of the IMD pathway. Furthermore, our analyses reveal that *miR-34* is transcriptionally repressed by the ecdysone signaling cascade in a manner that is dependent on the ecdysone receptor and the transcription factor BrC. Moreover, we characterize hormone-responsive *cis*-regulatory regions in the *miR-34* locus and *trans*-acting transcription factors required for ecdysone-mediated repression of *miR-34*. Lastly, we show that *miR-34* represses the expression of a number of components in ecdysone signaling, including Eip74EF, Eip75B and BrC. Thus our study uncovers *miR-34* as a component of an ecdysone-dependent regulatory circuit that modulates IMD innate immunity signaling in *Drosophila*.

Ecdysone-mediated activation of the EIP genes is achieved mainly by robust transcriptional activation, as mRNAs encoding EIP proteins accumulate rapidly upon ecdysone treatment, followed by a massive increase in levels of the EIP proteins. *Eip74EF*, a validated *miR-34* target gene, is among such group of early response genes. In flies lacking *miR-34*, dys-regulated expression of *Eip74EF* is linked to accelerated aging in the brain and shortened lifespan [64]. Here we identify *Eip75B*, another early response gene, as a new target of *miR-34*. In addition, levels of the BrC protein, which is a transcriptional factor and component of ecdysone signaling, is also significantly reduced upon *miR-34* over-expression (Fig 4A and 4B). Of note, the *BrC* transcript harbors a predicted *miR-34* binding site, which could underlie *miR-34*-mediated repression [46, 68]. Thus, our study lends strong support to the notion that certain miRNAs can coordinately target multiple components of a given biological process to achieve effective regulation by adding *miR-34* to such collection of miRNAs [71]. In addition, considering that the *miR-34* gene itself is transcriptionally repressed by ecdysone signaling, our findings indicate that ecdysone signaling not only activates select EIP genes transcriptionally to boost levels of the corresponding mRNAs, but also maximize the protein output from these mRNAs by reducing levels of *miR-34*. Thus, our study suggests that the cross-regulation between ecdysone signaling and *miR-34* expression appears to constitute a positive feedback loop, which may facilitate to achieve appropriate levels of output from ecdysone signaling under various physiological conditions. For example, several ecdysone bursts occur during early stages of *Drosophila* development [77]. As a consequence, ecdysone signaling prevails and keeps *miR-34* levels low. This ensures optimal output from ecdysone signaling, which is crucial for many aspects of development. In fact, flies lacking *miR-34* develop normally, consistent with the observed low levels of *miR-34* expression during early stages of development [64]. In contrast, levels of *miR-34* increase drastically in newly hatched adults and display a further elevation with age [64]. Coincidently ecdysone levels decline during pupae-adult transition and remain low in adult flies [77]. Thus it appears that in adult flies, *miR-34* expression prevails and further represses components of ecdysone signaling. Such repression of ecdysone signaling is physiologically relevant to adult flies. In fact, *miR-34* knockout flies display an early onset of neuro-degeneration during aging compared with age-matched wildtype counterparts. This phenotype is at least in part, attributable to elevated levels of Eip74EF, which on the one hand plays a key role in ecdysone signaling during early stages of fly development, and on the other hand, seems to become detrimental in adults [64].

**Fig 7:**
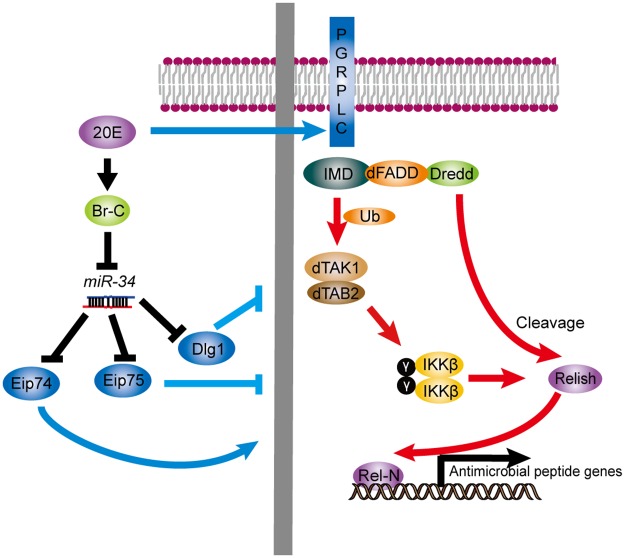
A schematic summary of the role of *miR-34* in modulating IMD innate immunity signaling.

While it is clear that ecdysone treatment primes S2 cells to become competent in innate immunity signaling, it appears that the underlying molecular mechanism is rather complex (Fig 7). For example, ecdysone signaling strongly activates the expression of *Eip74EF* and *Eip75B*, encoding transcription factors that operate as an activator and repressor of innate immunity signaling, respectively [33, 72]. In addition, with respect to miRNAs, ecdysone treatment markedly activates and inhibits, respectively, the expression of the miRNA genes *let-7* and *miR-34* [63]. Considering a previous report showing that *let-7* represses *Diptericin* [59] and the pro-immunity role of *miR-34* uncovered in this study, it would have been expected that ecdysone would reduce the output of the IMD innate immunity signaling pathway. Furthermore, our findings showing that *miR-34* can repress multiple genes of ecdysone signaling, including *Eip74EF*, *Eip75B* and *BrC*, add an additional layer of complexity. It is possible that these events take place at different phases of innate immunity signaling (e.g. initiation, maintenance and post-induction repression), reminiscent of the regulation of Pirk, which is activated at early phase of IMD signaling and represses the IMD pathway at a later phase [40–42]. Lastly, the newly identified *miR-34* target genes further expand the repertoire of potential negative regulators of IMD signaling. These genes display discrete expression profiles during development and immune activation, as they are likely subject to additional regulatory mechanisms besides *miR-34*. At any given time point, the output of innate immunity and ecdysone signaling is the net sum of outcomes from multiple regulatory processes. This may facilitate to achieve optimal outputs of innate immunity and ecdysone signaling during development and/or upon immune challenge. The precise sequence and interaction of events during the intricate interplay between ecdysone signaling and innate immunity during development, as well as the underlying mechanism remain to be determined.

Beside *Eip75B*, our study also identifies *dlg1* as another *miR-34* target gene relevant to innate immunity signaling. We show that 1) Dlg1 depletion resembles the phenotype elicited by *miR-34* over-expression; 2) *miR-34* over-expression causes a marked reduction in both mRNA and protein levels of Dlg1; 3) the 3’ UTR of the *dlg1* mRNA contains a functional *miR-34*-responsive site; and 4) Over-expression of *dlg1* abrogates the immune-stimulatory activity of *miR-34*. Dlg1 is localized to septate junctions and is a member of membrane-associated guanylate kinase (MAGUK) family proteins. Our findings are consistent with a recent study showing that the *big bang* (*bbg*) gene, which encodes a PDZ domain-containing protein that is present at the septate junctions, is required for gut epithelial barrier integrity. Flies homozygous for mutations in the *bbg* gene display constitutive activation of AMP genes in the gut by residential gut microbes [78]. Given that loss of *dlg1* in flies correlates with gut epithelial barrier dysfunction [79], and that knockdown of *dlg1* in mammals can attenuate the gut barrier integrity [80], an attractive possibility is that loss of *dlg1* in flies could lead to gut epithelial barrier dysfunction and constitutive expression of AMP genes due to leakage of gut microbes. In addition, our observations that Dlg1 also modulates the IMD pathway in cultured S2 cells also suggest a signaling role of Dlg1. For example, the mammalian scaffold protein CARMA1/CARD11, a member of the MAGUK family proteins, has been implicated in NF-κB signaling. In particular, CARD11 forms a complex with the adaptor protein BCL10 and the paracaspase MALT1 and mediates the activation of the NF-κB pathway upon T-cell activation [81, 82]. However, our attempt to uncover interactions between Dlg1 and canonical components of the IMD signaling pathway, including IMD and Kenny, was unsuccessful. Of note, since the *miR-34* effect on immunity is PGRP-LC-dependent, it would be interesting to assess potential interactions between Dlg1 and PGRP-LC. The molecular mechanism by which Dlg1 modulates innate immunity signaling warrants further investigation.

We noticed that levels of the Dlg1 protein display a steady decrease in S2 cells upon PGN treatment (S10 Fig). In addition, consistent with the observation made in S2 cells, we also detected a gradual decrease in levels of the Dlg1 protein over time in *E*. *coli*-infected flies (S11A Fig). Of note, levels of *miR-34* remain unchanged in *E*. *coli*-infected flies (S11B and S11C Fig), indicating that the observed decrease in Dlg1 protein levels is attributable to a *miR-34*-independent mechanism. Moreover, we detected a general reduction in *dlg1* mRNA levels upon depletion of canonical components of the IMD signaling pathway in S2 cells (S12 Fig). Lastly, a moderate drop in levels of the *dlg1* mRNA was detected at early stages of ecdysone treatment in S2 cells (S13 Fig). Taken together, these analyses suggest that the regulation of *dlg1* gene expression is mediated by both *miR-34*-dependent and *miR-34*-independent mechanisms.

We found that RNAi-mediated silencing of *Su(Z)12*, another predicted *miR-34* target gene [83], led to an elevation of *Diptericin* mRNA levels (S14 Fig). Thus, it appears that *miR-34* modulates innate immunity signaling by repressing multiple target genes. Our study joins other studies to support the notion that the function of a given miRNA is generally dictated not by a single, but rather by a cohort of target genes, and that the net biological outcome is reflected as a collective effect of multiple target genes.

Expression of *miR-34* is subject to complex regulation during the fly life cycle. *miR-34* is expressed at relatively low levels during early stages of development and is strongly repressed by ecdysone signaling at larval and pupal stages. Strikingly, *miR-34* levels are significantly elevated in adult flies upon eclosure and with age [64]. However, activation of IMD signaling does not appear to strongly affect *miR-34* expression, as levels of *miR-34* remain essentially unchanged in the body of flies upon *E*. *coli* infection (S11B and S11C Fig). Considering previous reports showing that innate immunity signaling is activated under various stress conditions and in aged flies, and that levels of *Diptericin* display an increase during aging [65–67], it is possible that elevated *miR-34* expression in aged flies may contribute to an increase in stress, which in turn leads to activation of innate immunity signaling pathways. Alternatively, *miR-34* may effect IMD signaling by repressing the afore-mentioned cohort of target genes, including *dlg1*, *Eip75B* and *Su(Z)12*, which encode negative regulators of IMD signaling. These two scenarios are not mutually exclusive.

Lastly, our study reveals that ecdysone signaling represses *miR-34* by transcriptional inhibition. We identify a *cis*-regulatory region (encompassing F3, Fig 6E) from the *miR-34* locus required for optimal ecdysone-mediated repression of *miR-34*. Interestingly, the identified genomic element coincides with the region that displays a high degree of occupancy by BrC, an ecdysone-induced transcription factor required for the optimal repression of *miR-34* by ecdysone. These observations suggest that BrC may directly contribute to ecdysone-mediated repression of *miR-34* by binding to the F3 region. Of note, three additional transcription factors, including Srp, Twi and Ap, also contribute to ecdysone-mediated repression of *miR-34*, and predicted Srp- and Twi-binding sites can be found within and close to F3, respectively (Fig 6E). It remains to be determined whether they directly bind to the F3 region.

In summary, our study identifies the conserved miRNA *miR-34*, together with several other miRNAs, as modulators of innate immunity in *Drosophila*. In particular, we show that *miR-34* achieves its immune-modulating function, at least in part, by repressing the expression of two novel target genes (*Dlg1* and *Eip75B*). In addition, our study reveals a mutual repression between *miR-34* expression and ecdysone signaling, and identifies *miR-34* as a crucial node in the intricate interplay between ecdysone signaling and innate immunity. Lastly, we identify key *cis*-regulatory genomic elements and *trans*-acting transcription factors required for optimal ecdysone-mediated repression of *miR-34*. Taken together, our study enriches the repertoire of immune-modulating miRNAs in animals, and provides new insights into the interplay between steroid hormone signaling and innate immunity.

## Materials and Methods

### Statistical analyses

All statistical analyses in this manuscript were performed using biological replicates and the sample number (n) is shown for each dataset in the corresponding legend. Most analyses were performed using student *t*-test, except for survival experiments, which involved the log rank test. Unless noted otherwise, data is shown in this manuscript as mean + standard errors of the mean (SEM), * *p*<0.05; ** *p*<0.01; *** *p*<0.001; **** *p*<0.0001.

### DNA constructs and antibodies

Dlg1 cDNA expression construct: a DNA fragment encoding Flag-Dlg1 was amplified by PCR and cloned into pRmHa-3 using SacI and BamHI restriction sites. Reporter construct carrying the UTR sequences from the *dlg1* cDNA: a *dlg1* 3’ UTR fragment was amplified by PCR and cloned into pRmHa-3-Renilla [84] using BamHI and SalI sites. To generate mutations in the candidate *miR-34*-binding site, the afore-mentioned reporter construct was subject to site-directed mutagenesis using Phusion Site-Directed Mutagenesis Kit (Thermo Fisher) according to the manufacturer’s instructions. Reporter constructs containing candidate *miR-34*-binding sites identified in the *Eip75B* ORF: pairs of oligos containing triple copies of individual sites (wildtype or mutant) were annealed and cloned into pRmHa-3-Renilla using BamHI and SalI sites. To generate various *miR-34* enhancer-*firefly luciferase* plasmid, Selected regions were PCR amplified from genomic DNA, cloned into pCR8-TOPO-GW (Invitrogen), and shuttled to pGL3_GW_luc+ [76] by LR clonase II recombination (Invitrogen). Expression construct for *miR-34*: a ~500 bp DNA fragment encoding *pri-miR-34* was amplified by PCR and cloned into pRmHa-3 using EcoRI and BamHI restriction sites. All constructs were verified by Sanger sequencing. The pMT-Eip75B-PC expression construct was a kind gift from Dr. Edward Dubrovsky [85]. Antibodies employed in this study include Anti-Eip74EF and anti-Eip75B antibodies (gift from Dr. Carl Thummel), anti-Dlg1 and anti-BrC antibodies (Hybridoma Bank).

### dsRNA synthesis

Templates for generating dsRNAs were either requested from the DRSC (flyRNAi.org) or amplified by PCR in house. dsRNAs were synthesized using Megascript *in vitro* transcription kit (Ambion) and purified using RNeasy column (Qiagen).

### *Drosophila* genetics and infection

Fly stocks are maintained on a standard fly food. To generate flies over-expressing select miRNAs, we crossed UAS-miRNA transgenic lines with the *da-Gal4; tub-Gal80* ^*ts*^ composite line. A control cross was set up between a shRNA transgenic line, which expresses an artificial sh-*gfp* RNA embedded in the *miR-1* cassette. To minimize lethality at early developmental stages due to the requirement for select miRNAs in development, fly crosses were kept at permissive temperature (18°C) until adult progeny of the appropriate genotype emerge. Subsequently the progeny was shifted to restrictive temperature (29°C) for 5 days to allow for miRNA transgene expression. For *in vivo* knockdown experiments, a similar crossing scheme was employed except that the UAS-shRNA lines were used instead of the UAS-miRNA lines. The *npr6* allele of *BrC* (BDSC stock #36562) was employed in experiments in Fig 5. For fly infection experiments, male progeny of the appropriate genotype were either left untreated (as control) or pricked with a sharp needle previously dipped in a concentrated suspension of either the Gram-negative bacteria *E*. *coli* or the Gram-positive bacteria *M*. *Luteus*, and flies were harvested 6 and 24 hours post-infection, respectively. Levels of mRNAs encoding the antimicrobial peptide genes *Diptericin* and *Drosomycin*, and the control *RpL32* mRNA were analyzed by RT-qPCR. Additional infection experiments were carried out by injecting 9.2 nl of bacterial suspension or PBS (control) into flies using Nanoject II (Drummond). For fly survival experiment, a concentrated culture of *Ecc15* (OD_600_ ~5); or a concentrated culture of *Enterobacter cloacae* (OD_600_ ~5) were injected. Fly survival was monitored daily. To determine pathogen load, a concentrated culture of *Ecc15* (OD_600_ ~5) or an overnight culture of *Enterobacter cloacae* (OD_600_ ~0.5) were injected into flies. Subsequently groups of 4 flies were harvested at various time points post *Enterobacter cloacae* infection (groups of 1 to 3 flies for *Ecc15* infection) and homogenized in 200 μl of sterile PBS. Diluted fly homogenates were plated onto LB plates containing nalidixic acid (Sigma) (for *Enterobacter cloacae*) or Ampicillin (for *Ecc15*), colony forming units (CFU) were recorded after 24 hours and CFU per fly is calculated. In experiments involving flies reared in antibiotics-containing food, flies were reared in standard fly food supplemented with antibiotics (100 μg/mL each of ampicillin, kanamycin and doxycycline).

### Bacterial phagocytosis assay

The assays were performed as previously described [86]. Briefly, 138 nl of pHrodo Red *E*. *coli* BioParticles Conjugate (Life Technologies) suspension in PBS was injected into individual flies of appropriate genotype using Nanoject II. Flies were kept at 25°C for 2 hours before imaging using an Axioskop Zeiss microscope.

### Cell culture, transfection, and RNAi

*Drosophila* S2 cells are maintained in Schneider’s medium (Invitrogen) supplemented with 10% fetal bovine serum (FBS) and 1% penicillin-streptomycin (Invitrogen). S2 cells stably expressing *miR-34* were generated by transfection with pRmHa-3-*miR-34* and the selection marker plasmid pHS-neo using the calcium phosphate method, followed by selection in medium containing 400 μg/mL G418 (Calbiochem). dsRNA treatment was performed as described previously [87–89]. Briefly, ~2 × 10^6^ S2 cells were seeded in 6-well plates for 24 h and then transfected with 3 μg of the appropriate dsRNA using the calcium phosphate protocol. Two days later, the cells were harvested, replated in 6-cm plates for 24 h, and then transfected again with another 9 μg of dsRNA. Three days later, the cells were harvested and used in assays. DNA transfection was preformed in the same manner as dsRNA transfections, except that only a single round of transfection was performed.

### Peptidoglycan and ecdysone treatment in S2 cells

*Drosophila* S2 cells were treated with 1 μM 20-hydroxyecdysone (Sigma) for 24 hours. Subsequently cells were treated with 10 μg/mL crude LPS prep (Sigma), which contains peptidoglycan (PGN) as a potent inducer of IMD signaling [6], for another 6 hours. Total RNA was extracted and levels of the antimicrobial peptide genes *Diptericin*, *Cecropin*, *Attacin A*, *Defensin*, *Metchnikowin* and *Drosomycin*, and the control *RpL32* mRNA were analyzed by RT-qPCR. For *miR-34* overexpression experiments, cells were first transfected with a *miR-34* expression construct controlled by the *metallothionein* promoter. One day post-transfection cells were treated with CuSO_4_ (250 μM) for 2 days prior to 20-hydroxyecdysone and PGN treatment.

### Reporter assay

For luciferase reporter assays, transfections were performed in a 96-well format and the amount of cells and DNA constructs were scaled down accordingly. Briefly, ~5 × 10^5^ S2 cells were seeded in 24-well plates the day before transfection. For *miR-34* target site reporter assays, 150 ng of the Renilla luciferase reporter gene containing wild type or mutant *miR-34*-binding site, 25 ng of a firefly luciferase reporter construct [84], and 600 ng of either the *miR-34* expression construct or the empty vector were transfected into these cells. Two days later, cells were treated with 250 μM copper sulfate for 24 hours and the reporter activity was measured using the Dual-Glo system (Promega). For data processing, *Renilla*/firefly ratio was calculated and normalized against the control sample (wildtype reporter in the absence of *miR-34*).

To identify the regulatory elements required for ecdysone-mediated repression of *miR-34*, 100 ng of *miR-34* enhancer-*firefly luciferase* plasmid and 100 ng of *pol III-Renilla luciferase* or 20 ng of *actin-Renilla luciferase* were transfected into cells. Two days later, cells were treated with 1 μM 20-hydroxyecdysone (Sigma) for 24 hours and luciferase activity was measured. For data processing, first, the firefly/*Renilla* ratio was calculated; next changes in the F/R ratio in cognate set of samples upon 20-HE treatment [F/R ratio _post 20-HE_ / F/R ratio _no 20-HE_] was calculated and normalized to the control sample (transfected with a firefly luciferase reporter gene driven by the regulatory region derived from the *traffic jam* gene, which is not responsive to ecdysone treatment).

### Co-immunoprecipitation

Various combinations of expression constructs for Flag-Dlg1, together with T7-IMD or T7-Kenny were transfected into S2 cells. Two days post-transfection, cells were treated with 250 μM CuSO4 to induce transgene expression and harvested another 24 hrs later. Cells were lysed in lysis buffer (20 mM Tris-HCl (pH 7.6), 150 mM NaCl, 2 mM EDTA, 10% glycerol, 1% Triton X-100, 1 mM DTT, 1 mM orthovanadate) supplemented with protease inhibitor cocktail (Roche). Cleared total lysates were immunoprecipitated with anti-Flag antibodies (Sigma). Both input and immunoprecipitated samples were analyzed by SDS-PAGE followed by immunoblotting with antibodies against the Flag (Sigma) or T7 (Novagen) epitopes.

### Chromatin immunoprecipitation (ChIP)

ChIP was performed as described previously with minor modifications [90]. Briefly, Cells were fixed by cross-linking with a final concentration of 1.42% formaldehyde for 15min at room temperature and then quenched with 125 mM glycine. After washing with cold PBS twice, the cells were lysed with IP buffer (150 mM NaCl, 5 mM EDTA, 0.5% NP-40, 1.0% Triton X-100, 50 mM Tris-HCl pH 7.5). The clarified lysate was subject to sonication. The chromatin was then sheared to fragments of 200–1000 bp and cleared of debris by centrifugation. The chromatin is used for ChIP using anti-BrC antibody (Hybridoma Bank). After washing in IP buffer, the precipitated ChIP DNA was eluted, the cross-links were reversed by incubation at 68°C for 2h (or overnight). DNA fragments then were purified from the isolated protein-DNA complex and analyzed by qPCR.

### RT-qPCR

Total cellular RNA was isolated with TRIzol (Invitrogen) and reverse transcription was carried out using QuantiTect Reverse Transcription kit (Qiagen). Quantitative PCR was performed using the iQ SYBR-green reagents on a CFX96 Real-Time PCR Detection System (Bio-Rad). The efficiency of various primer pairs was determined using serial dilutions of a standard template (RT samples from naïve or 20-HE treated S2 cells). Fold changes in RNA levels were calculated using the ΔΔCt method for primer pairs with efficiencies of 90–111%. Note that fold changes in the *dlg1* mRNA were determined by calculating absolute levels of *dlg1* and *RpL32* using the detected primer efficiency.

### Northern blotting

Northern blotting was performed as previously described [91, 92]. In brief, total cellular RNA was isolated with TRIzol (Invitrogen). Samples of 15 μg RNA were separated on 15% denaturing polyacrylamide gels and transferred to Hybond-N+ membranes (Amersham Biosciences) in 1X TBE buffer. Small RNAs were UV crosslinked to the membranes, and the membranes were prehybridized in hybridization buffer for 2 h. DNA probes complementary to the appropriate strands were 5′ radiolabeled and incubated with membranes overnight at 37°C. Membranes were washed twice in 1X SSC with 0.1% SDS at 42°C, and then exposed to Phosphorimager screens for 12–48 h. Membranes were stripped by the addition of boiling 0.1% SDS solution and incubated for 30 min.

### RNA-Seq data analysis

RNA-Seq datasets containing ~25 million 2 x 90 bp reads were generated from control S2 cells or cells over-expressing *miR-34* using an Illumina HiSeq 2000 system. The average insert size for the RNA-seq library was ~200 bp. Pair-end reads were mapped to the *D*. *melanogaster* genome (UCSC dm3) and to the Flock House virus genome using Bowtie2 (v2.1.0) with TopHat (v2.0.8b) [93]. Differentially expressed transcripts and genes were identified using Cufflinks (v2.1.1) [94].

### Oligonucleotides

See S3 Table.

## Supporting Information

S1 Fig Depletion of Drosha *in vivo* impacts innate immunity signaling. Total RNA was isolated from male flies carrying the ubiquitously expressed *da-Gal4* driver and a shRNA construct targeting Drosha (*da>Drosha shRNA*). Steady-state levels of mRNAs encoding Drosha (**A**), the AMPs Diptericin and Drosomycin (Drs), as well as levels of the primary *bantam* miRNA transcript (*pri-miR-bantam*) (**B**) were measured by qRT-PCR, and normalized to levels of the *RpL32* mRNA. RNA isolated from *da>gfp shRNA* males serves as negative control (n≥3; mean + SD).
(TIF)

S2 Fig Over-expression of *miR-34* dampens the Toll innate immunity signaling pathway. Flies over-expressing *miR-34* were infected by *M*. *luteus* via septic injury. After one day, RNA was extracted and levels of the *Drosomycin* (*Drs*) mRNA was measured by RT-qPCR and normalized to the control *RpL32* mRNA (n≥3).
(TIF)

S3 Fig Over-expression of *miR-34* in S2 cells impact AMP gene expression. A S2 cell line was established that stably expresses *miR-34* under the control of the copper-inducible *metallothionein* promoter. Cells were treated with ecdysone and a combination of CuSO_4_ (to activate the *metallothionein* promoter) and PGN. Total RNA was isolated and levels of mRNAs encoding various antimicrobial peptides, including Attacin A, Cecropin, Defensin and Metchnikowin were measured by RT-qPCR and normalized to the *RpL32* mRNA (mean + SD; n = 3).
(TIF)

S4 Fig Over-expression of *miR-34* in S2 cells activates *pirk* expression. The same set of RNA samples used in S3 Fig was subject to RT-qPCR analysis to examine levels of the *pirk* mRNA (mean + SD; n = 3).
(TIF)

S5 Fig *miR-34* overexpression or deficiency do not significantly impact bacterial phagocytosis. *miR-34* overexpression (*ox*) and knockout (*ko*) flies were injected with a suspension of pHrodo Red *E*. *coli* BioParticles Conjugate in PBS. Flies were kept at 25°C for 2 hours before imaging. Flies expressing *sh-gfp* (ctr) and *ko* flies carrying a *miR-34* rescue construct (*Res*), respectively, serve as controls. At least 9 flies per genotype were analyzed and a representative image from each genotype is shown.
(TIF)

S6 Fig Identification of candidate *miR-34* target genes. A Venn diagram shows the number of candidate *miR-34* target genes predicted by TargetScan or PicTar, as well as the number of genes that display a decrease in mRNA levels upon *miR-34* over-expression in S2 cell. A total of 27 genes were identified that scored positive in all three assays.
(TIF)

S7 Fig The *Eip75B* transcript harbors a second *miR-34*-binding site. (**A**) Reporter constructs were generated that carry either a wildtype (WT) or mutant (mut) *miR-34* binding site derived from the *Eip75B* ORF. Seed region of *miR-34* was highlighted in green. (**B**) The reporter constructs were transfected into S2 cells together with or without a *miR-34* expression construct, and reporter activities were measured (n = 3).
(TIF)

S8 Fig EcR and BrC are required for optimal ecdysone-mediated induction of *let-7* expression. S2 cells transfected with various dsRNAs were left untreated or treated with ecdysone (20-HE) at 1 μM for 48 hrs. Total RNA was isolated and levels of the primary *let-7* transcript were measured and normalized to the control *RpL32* mRNA (n = 3; mean + SD).
(TIF)

S9 Fig Knockdown of Srp, Twi and Ap. S2 cells transfected with various dsRNAs were left untreated or treated with ecdysone (20-HE) at 1 μM for 2 days. Total RNA was isolated and levels of the indicated transcripts were measured and normalized to the control *RpL32* mRNA (n = 3).
(TIF)

S10 Fig Levels of the Dlg1 protein display a decrease in response to IMD signaling in S2 cells. S2 cells were treated with 20-HE for 24 hrs prior to PGN treatment. Cells were harvested at different times post PGN treatment and levels of the Dlg1 protein were measured by immunoblot (**A**) and quantified (**B**; n = 3). The Tubulin protein serves as a loading control.
(TIF)

S11 Fig Levels of the Dlg1 protein display a decrease in response to IMD signaling *in vivo*. Flies were infected with *E*. *coli* using septic injury. Flies were harvested at different times and levels of the Dlg1 protein (**A**) and *miR-34* (**B**) in headless fly bodies were measured by immunoblot and Northern blot, respectively. The Tubulin protein and *2S* RNA serve as loading controls, respectively. (**C**) Levels of *miR-34* were quantified and normalized against the *2S* RNA (n = 3, mean + SD).
(TIF)

S12 Fig Levels of the *dlg1* mRNA display a decrease upon depletion of IMD signaling components in S2 cells. S2 cells were first treated with dsRNAs targeting various components of IMD signaling (below). Cells were subsequently treated with ecdysone for 24 hrs, and either remained untreated or treated with PGN for an additional 6 hours. Cells were harvested and levels of the *dlg1* mRNAs were measured by RT-qPCR and normalized against the *RpL32* mRNA. Results from two independent experiments are shown in **A** and **B**, respectively.
(TIF)

S13 Fig Levels of the *dlg1* mRNA display a decrease at early time points of ecdysone treatment in S2 cells. S2 cells were treated with ecdysone and harvested at different times. Levels of the *dlg1* and *Diptericin* mRNAs were measured by RT-qPCR and normalized to the control *RpL32* mRNA (n≥3).
(TIF)

S14 Fig *Suz12* is another *miR-34* target gene relevant to innate immunity signaling. S2 cells treated with a control dsRNA or dsRNA against *Suz12*. Subsequently, cells were either untreated or treated with PGN. Total RNA was isolated and levels of *Diptericin* were measured and normalized to the *RpL32* mRNA (n = 3).
(TIF)

S1 Table List of miRNAs analyzed for innate immunity phenotype. (PDF)

S2 Table List of 27 candidate *miR-34* target genes identified by TargetScan, PicTar and RNA-sequencing. Cells were transfected with various dsRNAs as indicated. After 3 days, cells were treated with ecdysone (20-HE) at 1 μM for an additional 24 hrs. Cells were harvested, total RNA was extracted and levels of *Diptericin* mRNA was measured by qPCR and normalized to *RpL32*. Cells transfected with a dsRNA against the firefly luciferase serve as a baseline control. Also shown are fold changes in the corresponding mRNA levels upon *miR-34* overexpression.
(PDF)

S3 Table Oligos employed in this study. (PDF)

## References

[1] Hetru C, Hoffmann JA. NF-kappaB in the immune response of Drosophila. Cold Spring Harb Perspect Biol. 2009;1(6):a000232 Epub 2010/05/12. 10.1101/cshperspect.a000232 20457557 PMC2882123

[2] Ramet M, Manfruelli P, Pearson A, Mathey-Prevot B, Ezekowitz RA. Functional genomic analysis of phagocytosis and identification of a Drosophila receptor for E. coli. Nature. 2002;416(6881):644–8. Epub 2002/03/26. 10.1038/nature735 11912489

[3] Gottar M, Gobert V, Michel T, Belvin M, Duyk G, Hoffmann JA, et al The Drosophila immune response against Gram-negative bacteria is mediated by a peptidoglycan recognition protein. Nature. 2002;416(6881):640–4. Epub 2002/03/26. 10.1038/nature734 11912488

[4] Choe KM, Lee H, Anderson KV. Drosophila peptidoglycan recognition protein LC (PGRP-LC) acts as a signal-transducing innate immune receptor. Proc Natl Acad Sci U S A. 2005;102(4):1122–6. Epub 2005/01/20. 10.1073/pnas.0404952102 15657141 PMC545828

[5] Choe KM, Werner T, Stoven S, Hultmark D, Anderson KV. Requirement for a peptidoglycan recognition protein (PGRP) in Relish activation and antibacterial immune responses in Drosophila. Science. 2002;296(5566):359–62. Epub 2002/03/02. 10.1126/science.1070216 11872802

[6] Kaneko T, Goldman WE, Mellroth P, Steiner H, Fukase K, Kusumoto S, et al Monomeric and polymeric gram-negative peptidoglycan but not purified LPS stimulate the Drosophila IMD pathway. Immunity. 2004;20(5):637–49. 15142531 10.1016/s1074-7613(04)00104-9

[7] Lemaitre B, Kromer-Metzger E, Michaut L, Nicolas E, Meister M, Georgel P, et al A recessive mutation, immune deficiency (imd), defines two distinct control pathways in the Drosophila host defense. Proc Natl Acad Sci U S A. 1995;92(21):9465–9. Epub 1995/10/10. 7568155 10.1073/pnas.92.21.9465 PMC40822

[8] Georgel P, Naitza S, Kappler C, Ferrandon D, Zachary D, Swimmer C, et al Drosophila immune deficiency (IMD) is a death domain protein that activates antibacterial defense and can promote apoptosis. Dev Cell. 2001;1(4):503–14. Epub 2001/11/13. 11703941 10.1016/s1534-5807(01)00059-4

[9] Leulier F, Rodriguez A, Khush RS, Abrams JM, Lemaitre B. The Drosophila caspase Dredd is required to resist Gram-negative bacterial infection. EMBO Rep. 2000;1(4):353–8. Epub 2001/03/28. 10.1093/embo-reports/kvd073 11269502 PMC1083747

[10] Leulier F, Vidal S, Saigo K, Ueda R, Lemaitre B. Inducible expression of double-stranded RNA reveals a role for dFADD in the regulation of the antibacterial response in Drosophila adults. Curr Biol. 2002;12(12):996–1000. Epub 2002/07/19. 12123572 10.1016/s0960-9822(02)00873-4

[11] Zhou R, Silverman N, Hong M, Liao DS, Chung Y, Chen ZJ, et al The role of ubiquitination in Drosophila innate immunity. J Biol Chem. 2005;280(40):34048–55. Epub 2005/08/06. 10.1074/jbc.M506655200 16081424

[12] Silverman N, Zhou R, Erlich RL, Hunter M, Bernstein E, Schneider D, et al Immune activation of NF-kappaB and JNK requires Drosophila TAK1. J Biol Chem. 2003;278(49):48928–34. Epub 2003/10/02. 10.1074/jbc.M304802200 14519762

[13] Kaneko T, Silverman N. Bacterial recognition and signalling by the Drosophila IMD pathway. Cell Microbiol. 2005;7(4):461–9. 10.1111/j.1462-5822.2005.00504.x 15760446

[14] Vidal S, Khush RS, Leulier F, Tzou P, Nakamura M, Lemaitre B. Mutations in the Drosophila dTAK1 gene reveal a conserved function for MAPKKKs in the control of rel/NF-kappaB-dependent innate immune responses. Genes Dev. 2001;15(15):1900–12. Epub 2001/08/04. 10.1101/gad.203301 11485985 PMC524699

[15] Silverman N, Zhou R, Stoven S, Pandey N, Hultmark D, Maniatis T. A Drosophila IkappaB kinase complex required for Relish cleavage and antibacterial immunity. Genes Dev. 2000;14(19):2461–71. Epub 2000/10/06. 11018014 10.1101/gad.817800 PMC316979

[16] Rutschmann S, Jung AC, Zhou R, Silverman N, Hoffmann JA, Ferrandon D. Role of Drosophila IKK gamma in a toll-independent antibacterial immune response. Nat Immunol. 2000;1(4):342–7. Epub 2001/03/23. 10.1038/79801 11017107

[17] Lu Y, Wu LP, Anderson KV. The antibacterial arm of the drosophila innate immune response requires an IkappaB kinase. Genes Dev. 2001;15(1):104–10. Epub 2001/01/13. 10.1101/gad.856901 11156609 PMC312606

[18] Stoven S, Ando I, Kadalayil L, Engstrom Y, Hultmark D. Activation of the Drosophila NF-kappaB factor Relish by rapid endoproteolytic cleavage. EMBO Rep. 2000;1(4):347–52. Epub 2001/03/28. 10.1093/embo-reports/kvd072 11269501 PMC1083746

[19] Stoven S, Silverman N, Junell A, Hedengren-Olcott M, Erturk D, Engstrom Y, et al Caspase-mediated processing of the Drosophila NF-kappaB factor Relish. Proc Natl Acad Sci U S A. 2003;100(10):5991–6. Epub 2003/05/07. 10.1073/pnas.1035902100 12732719 PMC156314

[20] Daigneault J, Klemetsaune L, Wasserman SA. The IRAK homolog Pelle is the functional counterpart of IkappaB kinase in the Drosophila Toll pathway. PLoS One. 2013;8(9):e75150 Epub 2013/10/03. 10.1371/journal.pone.0075150 24086459 PMC3781037

[21] Wasserman SA. Toll signaling: the enigma variations. Curr Opin Genet Dev. 2000;10(5):497–502. Epub 2000/09/12. 10980426 10.1016/s0959-437x(00)00118-0

[22] Meng X, Khanuja BS, Ip YT. Toll receptor-mediated Drosophila immune response requires Dif, an NF-kappaB factor. Genes Dev. 1999;13(7):792–7. Epub 1999/04/10. 10197979 10.1101/gad.13.7.792 PMC316597

[23] Rutschmann S, Jung AC, Hetru C, Reichhart JM, Hoffmann JA, Ferrandon D. The Rel protein DIF mediates the antifungal but not the antibacterial host defense in Drosophila. Immunity. 2000;12(5):569–80. Epub 2000/06/08. 10843389 10.1016/s1074-7613(00)80208-3

[24] Lemaitre B, Nicolas E, Michaut L, Reichhart JM, Hoffmann JA. The dorsoventral regulatory gene cassette spatzle/Toll/cactus controls the potent antifungal response in Drosophila adults. Cell. 1996;86(6):973–83. 8808632 10.1016/s0092-8674(00)80172-5

[25] Kurata S. Peptidoglycan recognition proteins in Drosophila immunity. Dev Comp Immunol. 2014;42(1):36–41. 10.1016/j.dci.2013.06.006 23796791 PMC3808481

[26] Bischoff V, Vignal C, Boneca IG, Michel T, Hoffmann JA, Royet J. Function of the drosophila pattern-recognition receptor PGRP-SD in the detection of Gram-positive bacteria. Nat Immunol. 2004;5(11):1175–80. 10.1038/ni1123 15448690

[27] Gobert V, Gottar M, Matskevich AA, Rutschmann S, Royet J, Belvin M, et al Dual activation of the Drosophila toll pathway by two pattern recognition receptors. Science. 2003;302(5653):2126–30. 10.1126/science.1085432 14684822

[28] Meister M. Blood cells of Drosophila: cell lineages and role in host defence. Curr Opin Immunol. 2004;16(1):10–5. 14734104 10.1016/j.coi.2003.11.002

[29] Thummel CS. Flies on steroids—Drosophila metamorphosis and the mechanisms of steroid hormone action. Trends Genet. 1996;12(8):306–10. Epub 1996/08/01. 8783940 10.1016/0168-9525(96)10032-9

[30] Dimarcq JL, Imler JL, Lanot R, Ezekowitz RA, Hoffmann JA, Janeway CA, et al Treatment of l(2)mbn Drosophila tumorous blood cells with the steroid hormone ecdysone amplifies the inducibility of antimicrobial peptide gene expression. Insect Biochem Mol Biol. 1997;27(10):877–86. 9474784 10.1016/s0965-1748(97)00072-6

[31] Flatt T, Heyland A, Rus F, Porpiglia E, Sherlock C, Yamamoto R, et al Hormonal regulation of the humoral innate immune response in Drosophila melanogaster. J Exp Biol. 2008;211(Pt 16):2712–24. 10.1242/jeb.014878 18689425 PMC2522372

[32] Zhang Z, Palli SR. Identification of a cis-regulatory element required for 20-hydroxyecdysone enhancement of antimicrobial peptide gene expression in Drosophila melanogaster. Insect Mol Biol. 2009;18(5):595–605. 10.1111/j.1365-2583.2009.00901.x 19754738

[33] Rus F, Flatt T, Tong M, Aggarwal K, Okuda K, Kleino A, et al Ecdysone triggered PGRP-LC expression controls Drosophila innate immunity. EMBO J. 2013;32(11):1626–38. Epub 2013/05/09. 10.1038/emboj.2013.100 23652443 PMC3671248

[34] Lanot R, Zachary D, Holder F, Meister M. Postembryonic hematopoiesis in Drosophila. Dev Biol. 2001;230(2):243–57. 10.1006/dbio.2000.0123 11161576

[35] Regan JC, Brandao AS, Leitao AB, Mantas Dias AR, Sucena E, Jacinto A, et al Steroid hormone signaling is essential to regulate innate immune cells and fight bacterial infection in Drosophila. PLoS Pathog. 2013;9(10):e1003720 10.1371/journal.ppat.1003720 24204269 PMC3812043

[36] Zhao JL, Rao DS, Boldin MP, Taganov KD, O'Connell RM, Baltimore D. NF-kappaB dysregulation in microRNA-146a-deficient mice drives the development of myeloid malignancies. Proc Natl Acad Sci U S A. 2011;108(22):9184–9. Epub 2011/05/18. 10.1073/pnas.1105398108 21576471 PMC3107319

[37] Aggarwal K, Silverman N. Positive and negative regulation of the Drosophila immune response. BMB Rep. 2008;41(4):267–77. 18452646 10.5483/bmbrep.2008.41.4.267

[38] Zaidman-Remy A, Herve M, Poidevin M, Pili-Floury S, Kim MS, Blanot D, et al The Drosophila amidase PGRP-LB modulates the immune response to bacterial infection. Immunity. 2006;24(4):463–73. 10.1016/j.immuni.2006.02.012 16618604

[39] Paredes JC, Welchman DP, Poidevin M, Lemaitre B. Negative regulation by amidase PGRPs shapes the Drosophila antibacterial response and protects the fly from innocuous infection. Immunity. 2011;35(5):770–9. 10.1016/j.immuni.2011.09.018 22118526

[40] Lhocine N, Ribeiro PS, Buchon N, Wepf A, Wilson R, Tenev T, et al PIMS modulates immune tolerance by negatively regulating Drosophila innate immune signaling. Cell Host Microbe. 2008;4(2):147–58. Epub 2008/08/12. 10.1016/j.chom.2008.07.004 18692774

[41] Kleino A, Myllymaki H, Kallio J, Vanha-aho LM, Oksanen K, Ulvila J, et al Pirk is a negative regulator of the Drosophila Imd pathway. J Immunol. 2008;180(8):5413–22. Epub 2008/04/09. 18390723 10.4049/jimmunol.180.8.5413

[42] Aggarwal K, Rus F, Vriesema-Magnuson C, Erturk-Hasdemir D, Paquette N, Silverman N. Rudra interrupts receptor signaling complexes to negatively regulate the IMD pathway. PLoS Pathog. 2008;4(8):e1000120 Epub 2008/08/09. 10.1371/journal.ppat.1000120 18688280 PMC2483946

[43] Kim M, Lee JH, Lee SY, Kim E, Chung J. Caspar, a suppressor of antibacterial immunity in Drosophila. Proc Natl Acad Sci U S A. 2006;103(44):16358–63. Epub 2006/10/20. 10.1073/pnas.0603238103 17050695 PMC1637587

[44] Tsichritzis T, Gaentzsch PC, Kosmidis S, Brown AE, Skoulakis EM, Ligoxygakis P, et al A Drosophila ortholog of the human cylindromatosis tumor suppressor gene regulates triglyceride content and antibacterial defense. Development. 2007;134(14):2605–14. 10.1242/dev.02859 17553907

[45] Tsuda M, Langmann C, Harden N, Aigaki T. The RING-finger scaffold protein Plenty of SH3s targets TAK1 to control immunity signalling in Drosophila. EMBO Rep. 2005;6(11):1082–7. 10.1038/sj.embor.7400537 16179944 PMC1371032

[46] Lewis BP, Shih IH, Jones-Rhoades MW, Bartel DP, Burge CB. Prediction of mammalian microRNA targets. Cell. 2003;115(7):787–98. Epub 2003/12/31. 14697198 10.1016/s0092-8674(03)01018-3

[47] Lai EC, Tam B, Rubin GM. Pervasive regulation of Drosophila Notch target genes by GY-box-, Brd-box-, and K-box-class microRNAs. Genes Dev. 2005;19(9):1067–80. Epub 2005/04/19. 10.1101/gad.1291905 15833912 PMC1091741

[48] Guo H, Ingolia NT, Weissman JS, Bartel DP. Mammalian microRNAs predominantly act to decrease target mRNA levels. Nature. 2010;466(7308):835–40. Epub 2010/08/13. 10.1038/nature09267 20703300 PMC2990499

[49] Pillai RS, Bhattacharyya SN, Artus CG, Zoller T, Cougot N, Basyuk E, et al Inhibition of translational initiation by Let-7 MicroRNA in human cells. Science. 2005;309(5740):1573–6. Epub 2005/08/06. 10.1126/science.1115079 16081698

[50] Bazzini AA, Lee MT, Giraldez AJ. Ribosome profiling shows that miR-430 reduces translation before causing mRNA decay in zebrafish. Science. 2012;336(6078):233–7. Epub 2012/03/17. 10.1126/science.1215704 22422859 PMC3547538

[51] Djuranovic S, Nahvi A, Green R. miRNA-mediated gene silencing by translational repression followed by mRNA deadenylation and decay. Science. 2012;336(6078):237–40. Epub 2012/04/14. 10.1126/science.1215691 22499947 PMC3971879

[52] Fabian MR, Mathonnet G, Sundermeier T, Mathys H, Zipprich JT, Svitkin YV, et al Mammalian miRNA RISC recruits CAF1 and PABP to affect PABP-dependent deadenylation. Mol Cell. 2009;35(6):868–80. Epub 2009/09/01. 10.1016/j.molcel.2009.08.004 19716330 PMC2803087

[53] Bushati N, Cohen SM. microRNA functions. Annu Rev Cell Dev Biol. 2007;23:175–205. Epub 2007/05/18. 10.1146/annurev.cellbio.23.090506.123406 17506695

[54] O'Connell RM, Taganov KD, Boldin MP, Cheng G, Baltimore D. MicroRNA-155 is induced during the macrophage inflammatory response. Proc Natl Acad Sci U S A. 2007;104(5):1604–9. Epub 2007/01/24. 10.1073/pnas.0610731104 17242365 PMC1780072

[55] Thai TH, Calado DP, Casola S, Ansel KM, Xiao C, Xue Y, et al Regulation of the germinal center response by microRNA-155. Science. 2007;316(5824):604–8. Epub 2007/04/28. 10.1126/science.1141229 17463289

[56] Taganov KD, Boldin MP, Chang KJ, Baltimore D. NF-kappaB-dependent induction of microRNA miR-146, an inhibitor targeted to signaling proteins of innate immune responses. Proc Natl Acad Sci U S A. 2006;103(33):12481–6. Epub 2006/08/04. 10.1073/pnas.0605298103 16885212 PMC1567904

[57] Fullaondo A, Lee SY. Identification of putative miRNA involved in Drosophila melanogaster immune response. Dev Comp Immunol. 2012;36(2):267–73. Epub 2011/06/07. 10.1016/j.dci.2011.03.034 21641929

[58] Choi IK, Hyun S. Conserved microRNA miR-8 in fat body regulates innate immune homeostasis in Drosophila. Dev Comp Immunol. 2012;37(1):50–4. Epub 2012/01/03. 10.1016/j.dci.2011.12.008 22210547

[59] Garbuzov A, Tatar M. Hormonal regulation of Drosophila microRNA let-7 and miR-125 that target innate immunity. Fly (Austin). 2010;4(4):306–11. Epub 2010/08/28. 20798594 10.4161/fly.4.4.13008 PMC3174482

[60] Lee Y, Ahn C, Han J, Choi H, Kim J, Yim J, et al The nuclear RNase III Drosha initiates microRNA processing. Nature. 2003;425(6956):415–9. Epub 2003/09/26. 10.1038/nature01957 14508493

[61] Ni JQ, Zhou R, Czech B, Liu LP, Holderbaum L, Yang-Zhou D, et al A genome-scale shRNA resource for transgenic RNAi in Drosophila. Nat Methods. 2011;8(5):405–7. Epub 2011/04/05. 10.1038/nmeth.1592 21460824 PMC3489273

[62] Bejarano F, Bortolamiol-Becet D, Dai Q, Sun K, Saj A, Chou YT, et al A genome-wide transgenic resource for conditional expression of Drosophila microRNAs. Development. 2012;139(15):2821–31. Epub 2012/06/30. 10.1242/dev.079939 22745315 PMC3392707

[63] Sempere LF, Sokol NS, Dubrovsky EB, Berger EM, Ambros V. Temporal regulation of microRNA expression in Drosophila melanogaster mediated by hormonal signals and broad-Complex gene activity. Dev Biol. 2003;259(1):9–18. Epub 2003/06/19. 12812784 10.1016/s0012-1606(03)00208-2

[64] Liu N, Landreh M, Cao K, Abe M, Hendriks GJ, Kennerdell JR, et al The microRNA miR-34 modulates ageing and neurodegeneration in Drosophila. Nature. 2012;482(7386):519–23. Epub 2012/02/22. 10.1038/nature10810 22343898 PMC3326599

[65] Zerofsky M, Harel E, Silverman N, Tatar M. Aging of the innate immune response in Drosophila melanogaster. Aging Cell. 2005;4(2):103–8. Epub 2005/03/18. 10.1111/j.1474-9728.2005.00147.x 15771614

[66] Pletcher SD, Macdonald SJ, Marguerie R, Certa U, Stearns SC, Goldstein DB, et al Genome-wide transcript profiles in aging and calorically restricted Drosophila melanogaster. Curr Biol. 2002;12(9):712–23. Epub 2002/05/15. 12007414 10.1016/s0960-9822(02)00808-4

[67] Seroude L, Brummel T, Kapahi P, Benzer S. Spatio-temporal analysis of gene expression during aging in Drosophila melanogaster. Aging Cell. 2002;1(1):47–56. Epub 2003/07/29. 12882353 10.1046/j.1474-9728.2002.00007.x

[68] Lewis BP, Burge CB, Bartel DP. Conserved seed pairing, often flanked by adenosines, indicates that thousands of human genes are microRNA targets. Cell. 2005;120(1):15–20. Epub 2005/01/18. 10.1016/j.cell.2004.12.035 15652477

[69] Krek A, Grun D, Poy MN, Wolf R, Rosenberg L, Epstein EJ, et al Combinatorial microRNA target predictions. Nat Genet. 2005;37(5):495–500. Epub 2005/04/05. 10.1038/ng1536 15806104

[70] Woods DF, Hough C, Peel D, Callaini G, Bryant PJ. Dlg protein is required for junction structure, cell polarity, and proliferation control in Drosophila epithelia. J Cell Biol. 1996;134(6):1469–82. Epub 1996/09/01. 8830775 10.1083/jcb.134.6.1469 PMC2120992

[71] Brennecke J, Stark A, Russell RB, Cohen SM. Principles of microRNA-target recognition. PLoS Biol. 2005;3(3):e85 Epub 2005/02/22. 10.1371/journal.pbio.0030085 15723116 PMC1043860

[72] Kleino A, Valanne S, Ulvila J, Kallio J, Myllymaki H, Enwald H, et al Inhibitor of apoptosis 2 and TAK1-binding protein are components of the Drosophila Imd pathway. EMBO J. 2005;24(19):3423–34. Epub 2005/09/16. 10.1038/sj.emboj.7600807 16163390 PMC1276168

[73] Thomas HE, Stunnenberg HG, Stewart AF. Heterodimerization of the Drosophila ecdysone receptor with retinoid X receptor and ultraspiracle. Nature. 1993;362(6419):471–5. Epub 1993/04/01. 10.1038/362471a0 8385270

[74] Yao TP, Forman BM, Jiang Z, Cherbas L, Chen JD, McKeown M, et al Functional ecdysone receptor is the product of EcR and Ultraspiracle genes. Nature. 1993;366(6454):476–9. Epub 1993/12/02. 10.1038/366476a0 8247157

[75] Shlyueva D, Stelzer C, Gerlach D, Yanez-Cuna JO, Rath M, Boryn LM, et al Hormone-responsive enhancer-activity maps reveal predictive motifs, indirect repression, and targeting of closed chromatin. Mol Cell. 2014;54(1):180–92. Epub 2014/04/02. 10.1016/j.molcel.2014.02.026 24685159

[76] Arnold CD, Gerlach D, Stelzer C, Boryn LM, Rath M, Stark A. Genome-wide quantitative enhancer activity maps identified by STARR-seq. Science. 2013;339(6123):1074–7. Epub 2013/01/19. 10.1126/science.1232542 23328393

[77] Handler AM. Ecdysteroid titers during pupal and adult development in Drosophila melanogaster. Dev Biol. 1982;93(1):73–82. Epub 1982/09/01. 6813165 10.1016/0012-1606(82)90240-8

[78] Bonnay F, Cohen-Berros E, Hoffmann M, Kim SY, Boulianne GL, Hoffmann JA, et al big bang gene modulates gut immune tolerance in Drosophila. Proc Natl Acad Sci U S A. 2013;110(8):2957–62. 10.1073/pnas.1221910110 23378635 PMC3581892

[79] Clark RI, Salazar A, Yamada R, Fitz-Gibbon S, Morselli M, Alcaraz J, et al Distinct Shifts in Microbiota Composition during Drosophila Aging Impair Intestinal Function and Drive Mortality. Cell Rep. 2015;12(10):1656–67. 10.1016/j.celrep.2015.08.004 26321641 PMC4565751

[80] Ivanov AI, Young C, Den Beste K, Capaldo CT, Humbert PO, Brennwald P, et al Tumor suppressor scribble regulates assembly of tight junctions in the intestinal epithelium. Am J Pathol. 2010;176(1):134–45. 10.2353/ajpath.2010.090220 19959811 PMC2797876

[81] Pomerantz JL, Denny EM, Baltimore D. CARD11 mediates factor-specific activation of NF-kappaB by the T cell receptor complex. EMBO J. 2002;21(19):5184–94. Epub 2002/10/03. 10.1093/emboj/cdf505 12356734 PMC129028

[82] Thome M, Charton JE, Pelzer C, Hailfinger S. Antigen receptor signaling to NF-kappaB via CARMA1, BCL10, and MALT1. Cold Spring Harb Perspect Biol. 2010;2(9):a003004 Epub 2010/08/06. 10.1101/cshperspect.a003004 20685844 PMC2926749

[83] Robins H, Li Y, Padgett RW. Incorporating structure to predict microRNA targets. Proc Natl Acad Sci U S A. 2005;102(11):4006–9. Epub 2005/03/02. 10.1073/pnas.0500775102 15738385 PMC554828

[84] Zhou R, Hotta I, Denli AM, Hong P, Perrimon N, Hannon GJ. Comparative analysis of argonaute-dependent small RNA pathways in Drosophila. Mol Cell. 2008;32(4):592–9. 10.1016/j.molcel.2008.10.018 19026789 PMC2615197

[85] Dubrovsky EB, Dubrovskaya VA, Berger EM. Hormonal regulation and functional role of Drosophila E75A orphan nuclear receptor in the juvenile hormone signaling pathway. Dev Biol. 2004;268(2):258–70. 10.1016/j.ydbio.2004.01.009 15063166

[86] Cuttell L, Vaughan A, Silva E, Escaron CJ, Lavine M, Van Goethem E, et al Undertaker, a Drosophila Junctophilin, links Draper-mediated phagocytosis and calcium homeostasis. Cell. 2008;135(3):524–34. 10.1016/j.cell.2008.08.033 18984163

[87] Xiong XP, Kurthkoti K, Chang KY, Lichinchi G, De N, Schneemann A, et al Core small nuclear ribonucleoprotein particle splicing factor SmD1 modulates RNA interference in Drosophila. Proc Natl Acad Sci U S A. 2013;110(41):16520–5. Epub 2013/09/27. 10.1073/pnas.1315803110 24067655 PMC3799365

[88] Xiong XP, Vogler G, Kurthkoti K, Samsonova A, Zhou R. SmD1 Modulates the miRNA Pathway Independently of Its Pre-mRNA Splicing Function. PLoS Genet. 2015;11(8):e1005475 Epub 2015/08/27. 10.1371/journal.pgen.1005475 26308709 PMC4550278

[89] Lim SJ, Scott A, Xiong XP, Vahidpour S, Karijolich J, Guo D, et al Requirement for CRIF1 in RNA interference and Dicer-2 stability. RNA Biol. 2014;11(9):1171–9. Epub 2014/12/09. 10.4161/rna.34381 25483042 PMC4615304

[90] Nelson JD, Denisenko O, Bomsztyk K. Protocol for the fast chromatin immunoprecipitation (ChIP) method. Nat Protoc. 2006;1(1):179–85. Epub 2007/04/05. 10.1038/nprot.2006.27 17406230

[91] Czech B, Malone CD, Zhou R, Stark A, Schlingeheyde C, Dus M, et al An endogenous small interfering RNA pathway in Drosophila. Nature. 2008;453(7196):798–802. Epub 2008/05/09. 10.1038/nature07007 18463631 PMC2895258

[92] Zhou R, Czech B, Brennecke J, Sachidanandam R, Wohlschlegel JA, Perrimon N, et al Processing of Drosophila endo-siRNAs depends on a specific Loquacious isoform. RNA. 2009;15(10):1886–95. Epub 2009/07/29. 10.1261/rna.1611309 19635780 PMC2743050

[93] Kim D, Pertea G, Trapnell C, Pimentel H, Kelley R, Salzberg SL. TopHat2: accurate alignment of transcriptomes in the presence of insertions, deletions and gene fusions. Genome Biol. 2013;14(4):R36 Epub 2013/04/27. 10.1186/gb-2013-14-4-r36 23618408 PMC4053844

[94] Trapnell C, Hendrickson DG, Sauvageau M, Goff L, Rinn JL, Pachter L. Differential analysis of gene regulation at transcript resolution with RNA-seq. Nat Biotechnol. 2013;31(1):46–53. Epub 2012/12/12. 10.1038/nbt.2450 23222703 PMC3869392

