# Supplementary figures and images for: miR-34 Modulates Innate Immunity and Ecdysone Signaling in Drosophila
Source: PLoS Pathog. 2016 Nov 28;12(11):e1006034. doi: 10.1371/journal.ppat.1006034 (PMC5125713; doi:10.1371/journal.ppat.1006034)

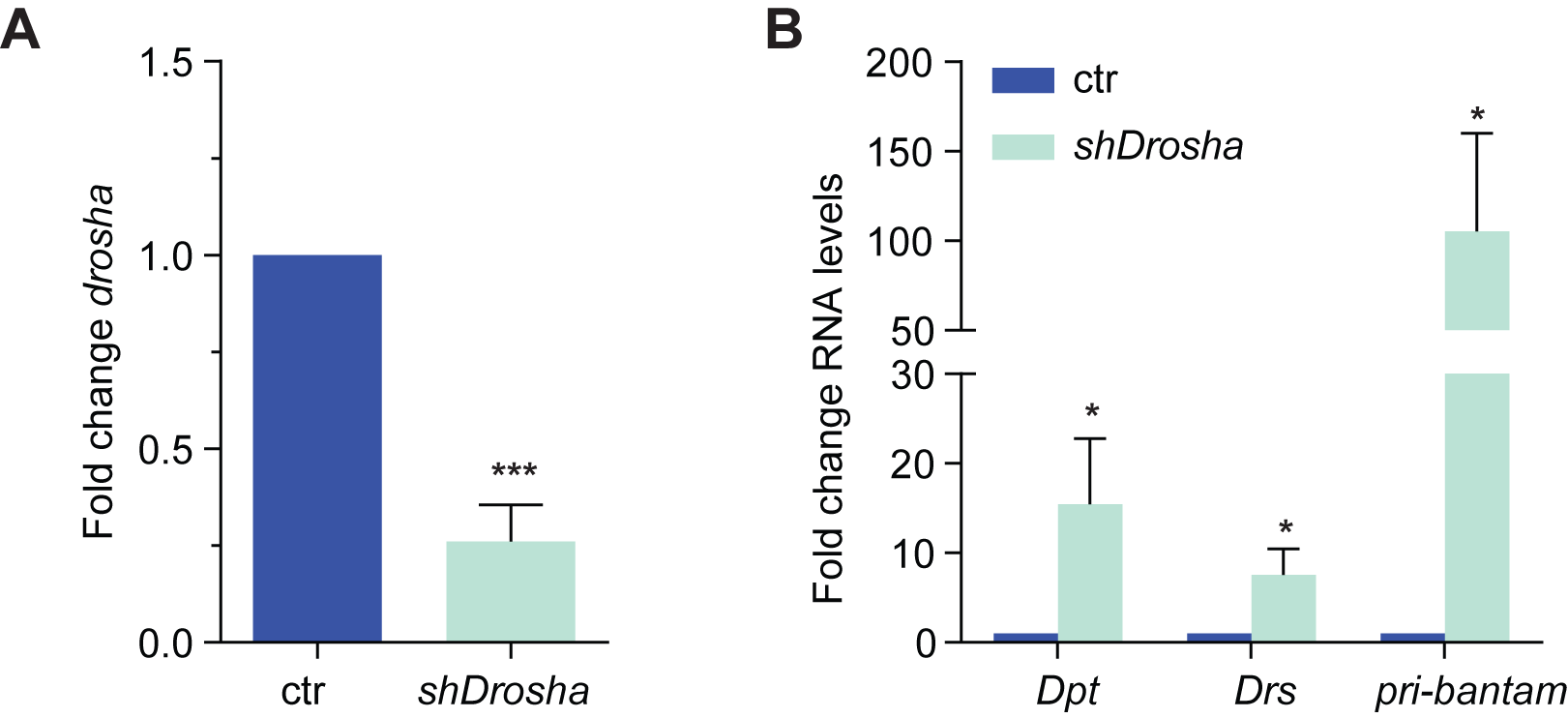

Supplement: S1 Fig — Total RNA was isolated from male flies carrying the ubiquitously expressed da-Gal4 driver and a shRNA construct targeting Drosha (da>Drosha shRNA). Steady-state levels of mRNAs encoding Drosha (A), the AMPs Diptericin and Drosomycin (Drs), as well as levels of the primary bantam miRNA transcript (pri-miR-bantam) (B) were measured by qRT-PCR, and normalized to levels of the RpL32 mRNA. RNA isolated from da>gfp shRNA males serves as negative control (n≥3; mean + SD). (TIF) [file ppat.1006034.s001.tif]

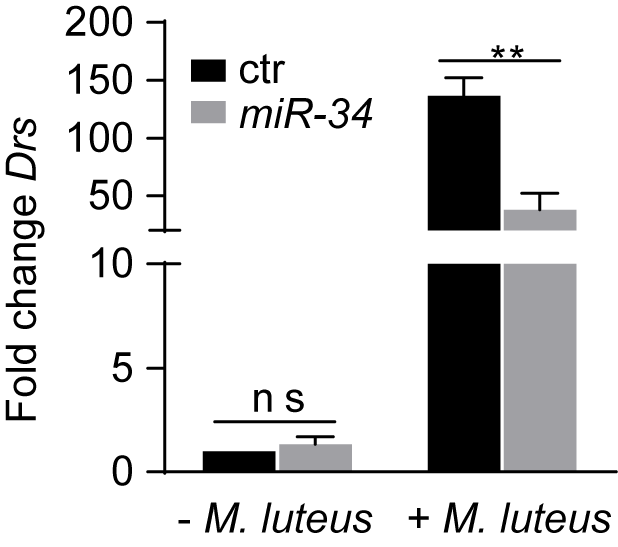

Supplement: S2 Fig — Flies over-expressing miR-34 were infected by M. luteus via septic injury. After one day, RNA was extracted and levels of the Drosomycin (Drs) mRNA was measured by RT-qPCR and normalized to the control RpL32 mRNA (n≥3). (TIF) [file ppat.1006034.s002.tif]

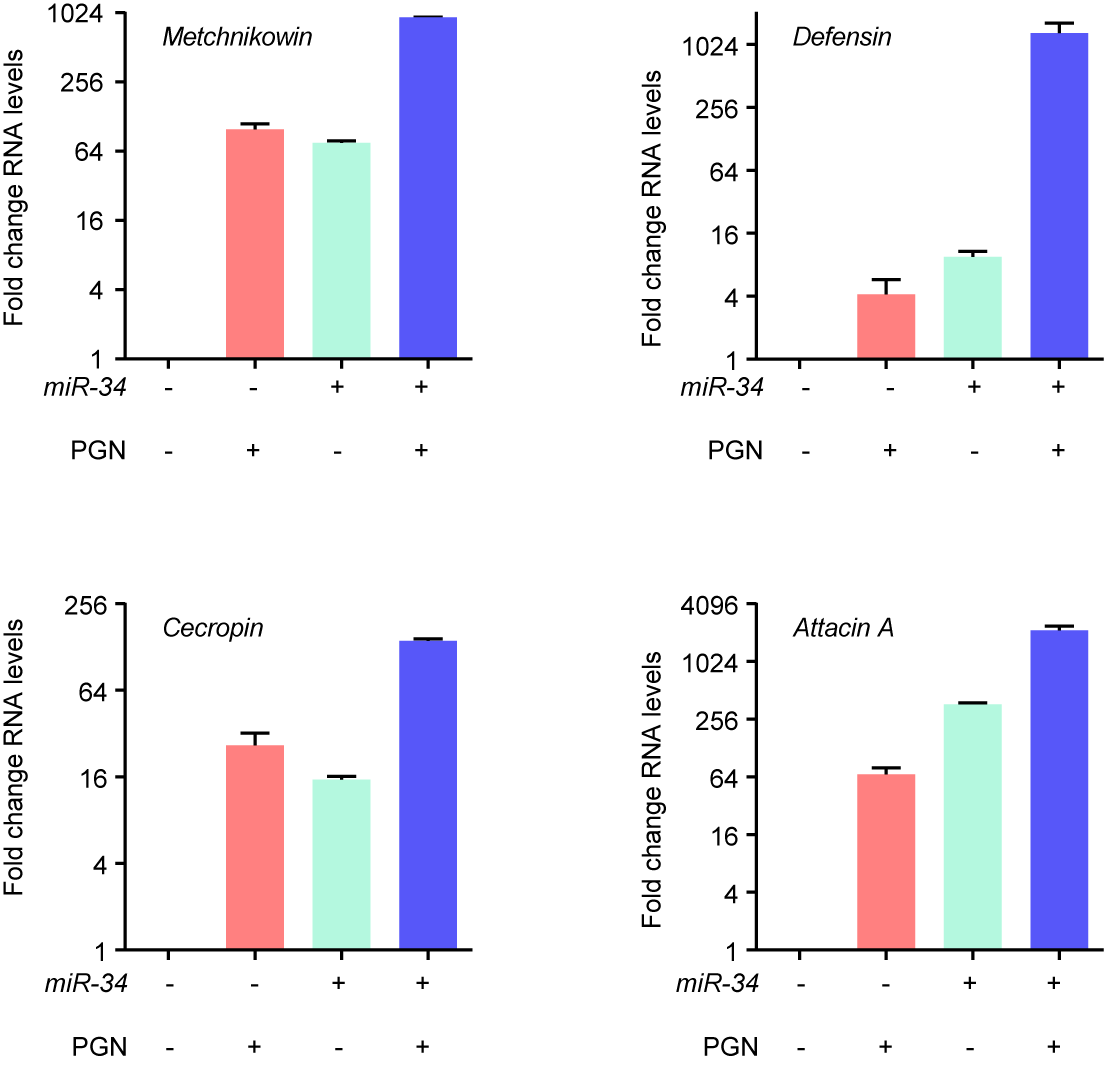

Supplement: S3 Fig — A S2 cell line was established that stably expresses miR-34 under the control of the copper-inducible metallothionein promoter. Cells were treated with ecdysone and a combination of CuSO4 (to activate the metallothionein promoter) and PGN. Total RNA was isolated and levels of mRNAs encoding various antimicrobial peptides, including Attacin A, Cecropin, Defensin and Metchnikowin were measured by RT-qPCR and normalized to the RpL32 mRNA (mean + SD; n = 3). (TIF) [file ppat.1006034.s003.tif]

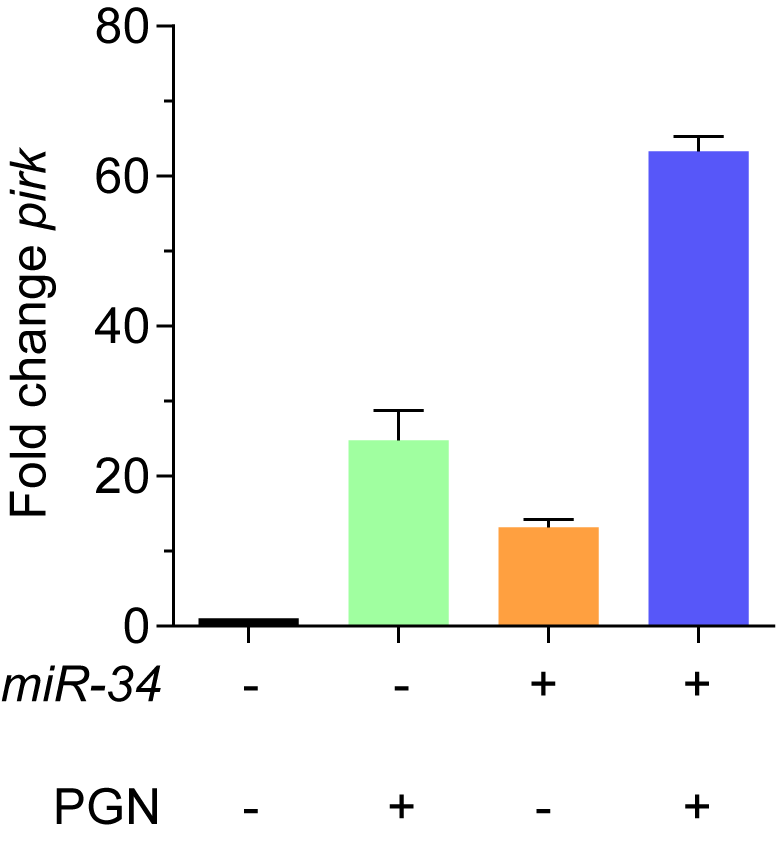

Supplement: S4 Fig — The same set of RNA samples used in S3 Fig was subject to RT-qPCR analysis to examine levels of the pirk mRNA (mean + SD; n = 3). (TIF) [file ppat.1006034.s004.tif]

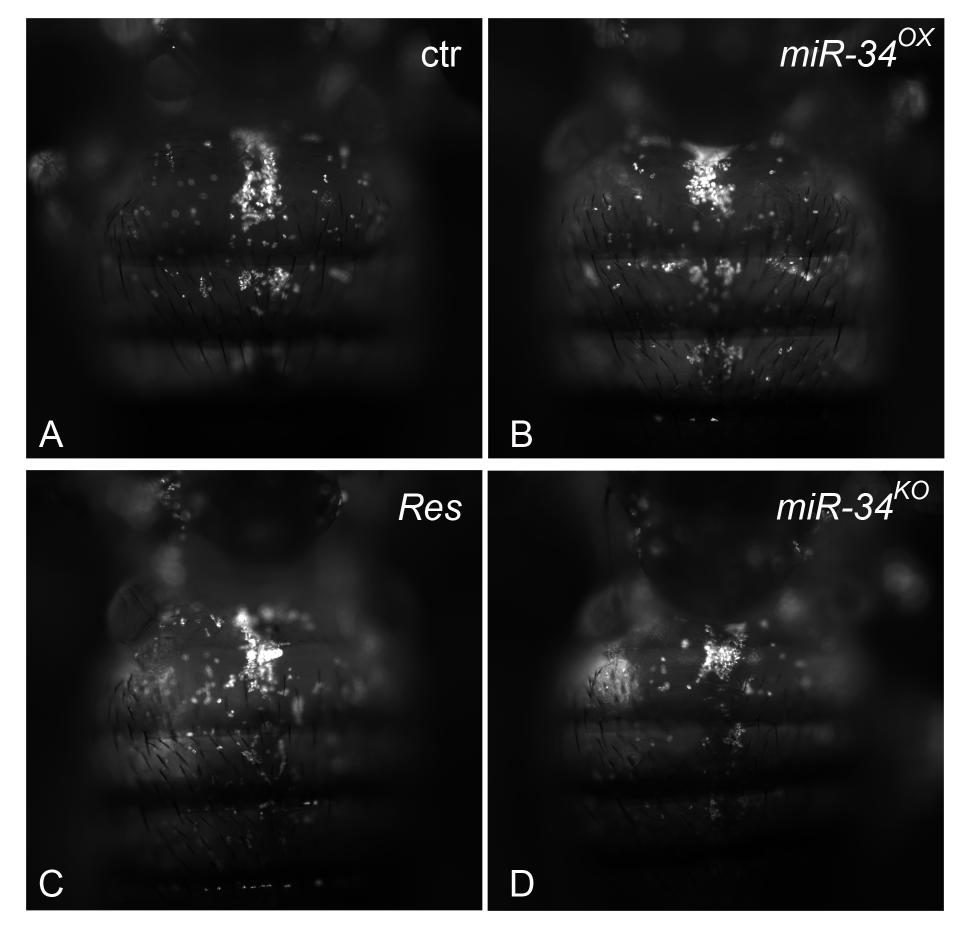

Supplement: S5 Fig — miR-34 overexpression (ox) and knockout (ko) flies were injected with a suspension of pHrodo Red E. coli BioParticles Conjugate in PBS. Flies were kept at 25°C for 2 hours before imaging. Flies expressing sh-gfp (ctr) and ko flies carrying a miR-34 rescue construct (Res), respectively, serve as controls. At least 9 flies per genotype were analyzed and a representative image from each genotype is shown. (TIF) [file ppat.1006034.s005.tif]

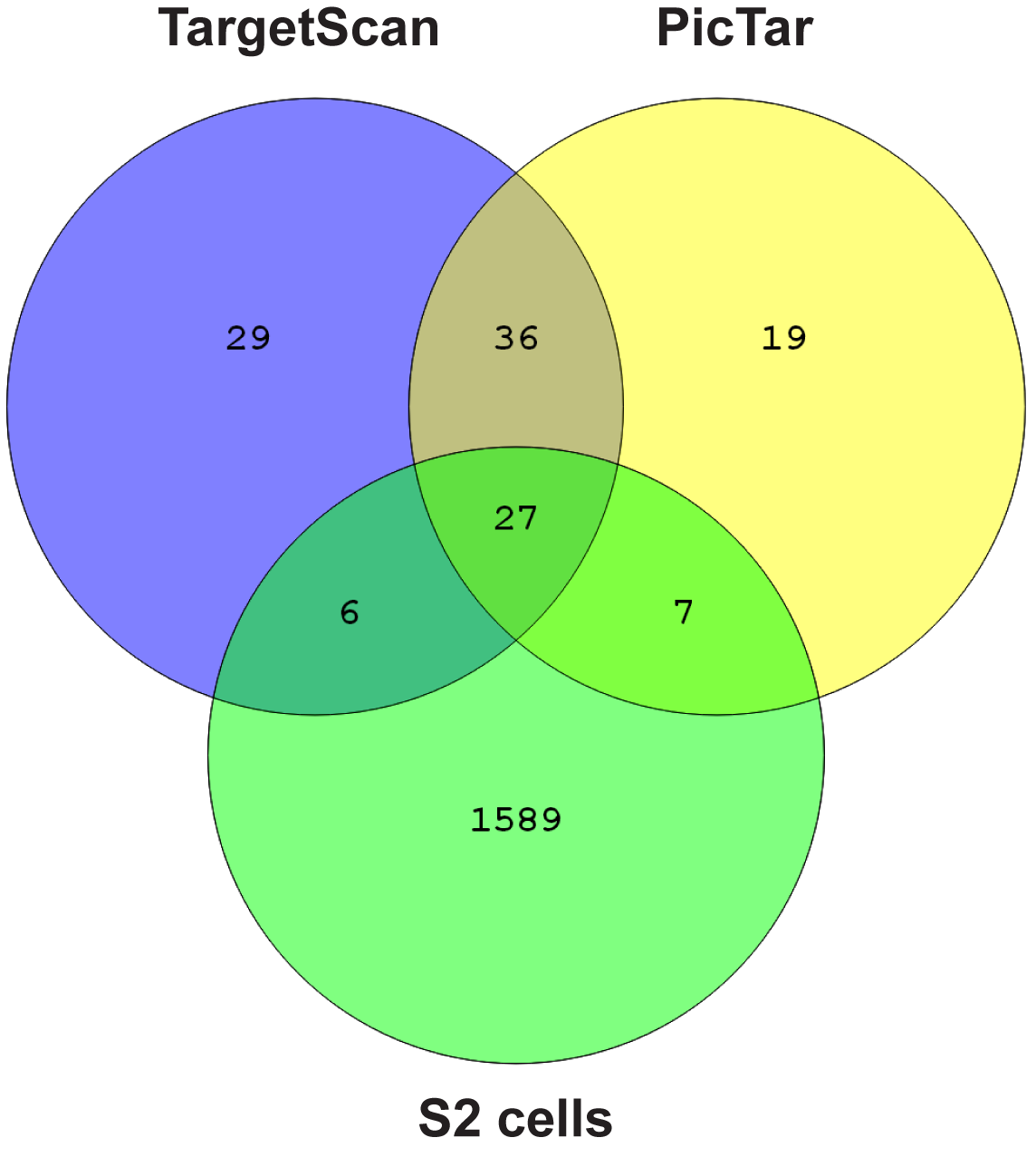

Supplement: S6 Fig — A Venn diagram shows the number of candidate miR-34 target genes predicted by TargetScan or PicTar, as well as the number of genes that display a decrease in mRNA levels upon miR-34 over-expression in S2 cell. A total of 27 genes were identified that scored positive in all three assays. (TIF) [file ppat.1006034.s006.tif]

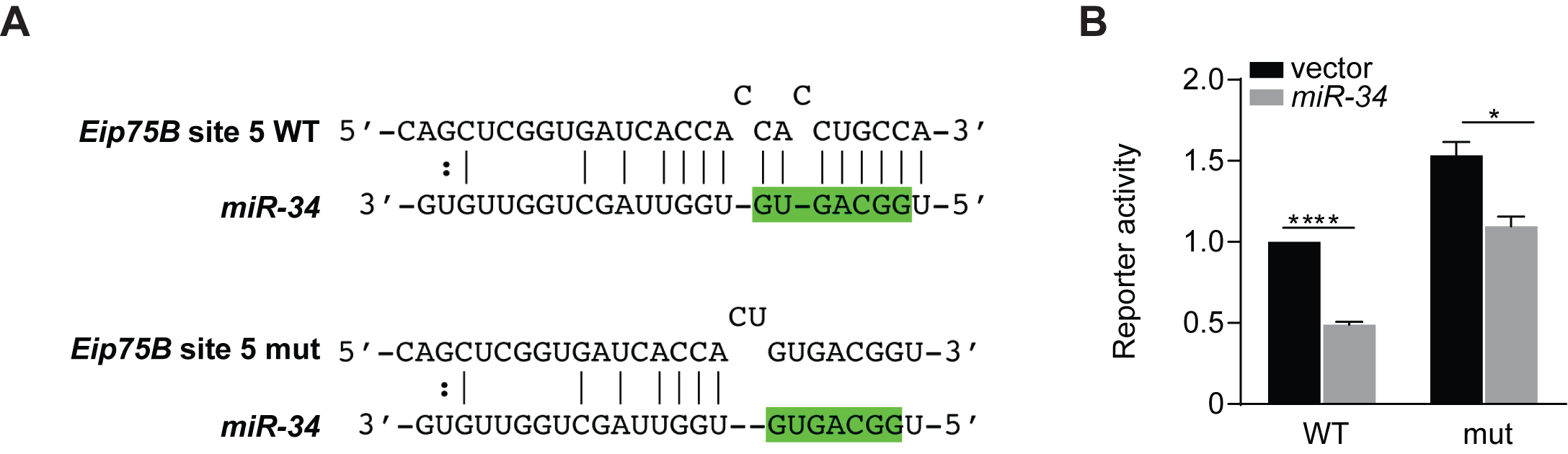

Supplement: S7 Fig — (A) Reporter constructs were generated that carry either a wildtype (WT) or mutant (mut) miR-34 binding site derived from the Eip75B ORF. Seed region of miR-34 was highlighted in green. (B) The reporter constructs were transfected into S2 cells together with or without a miR-34 expression construct, and reporter activities were measured (n = 3). (TIF) [file ppat.1006034.s007.tif]

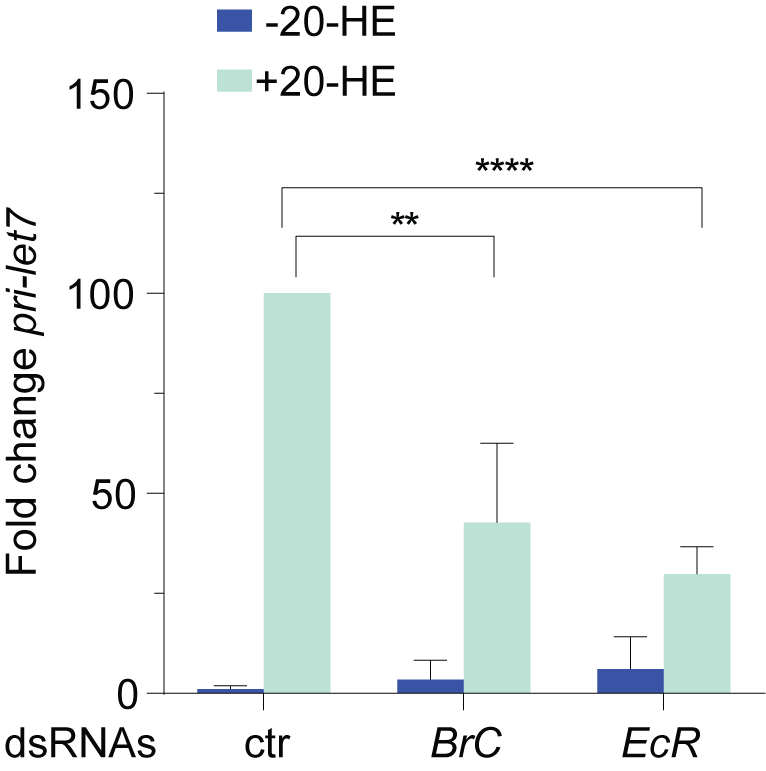

Supplement: S8 Fig — S2 cells transfected with various dsRNAs were left untreated or treated with ecdysone (20-HE) at 1 μM for 48 hrs. Total RNA was isolated and levels of the primary let-7 transcript were measured and normalized to the control RpL32 mRNA (n = 3; mean + SD). (TIF) [file ppat.1006034.s008.tif]

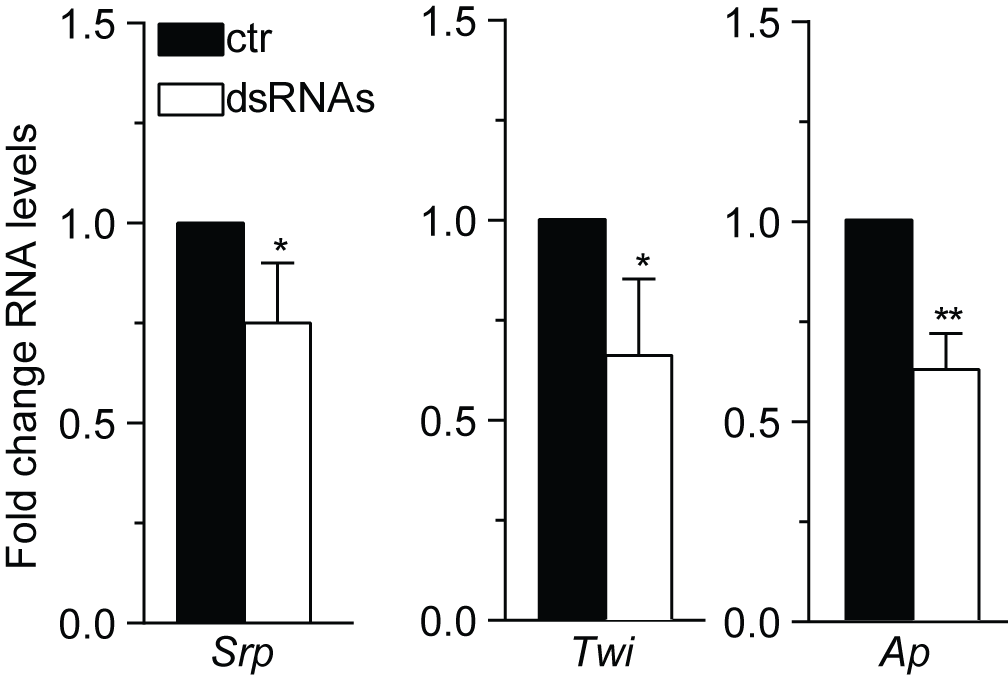

Supplement: S9 Fig — S2 cells transfected with various dsRNAs were left untreated or treated with ecdysone (20-HE) at 1 μM for 2 days. Total RNA was isolated and levels of the indicated transcripts were measured and normalized to the control RpL32 mRNA (n = 3). (TIF) [file ppat.1006034.s009.tif]

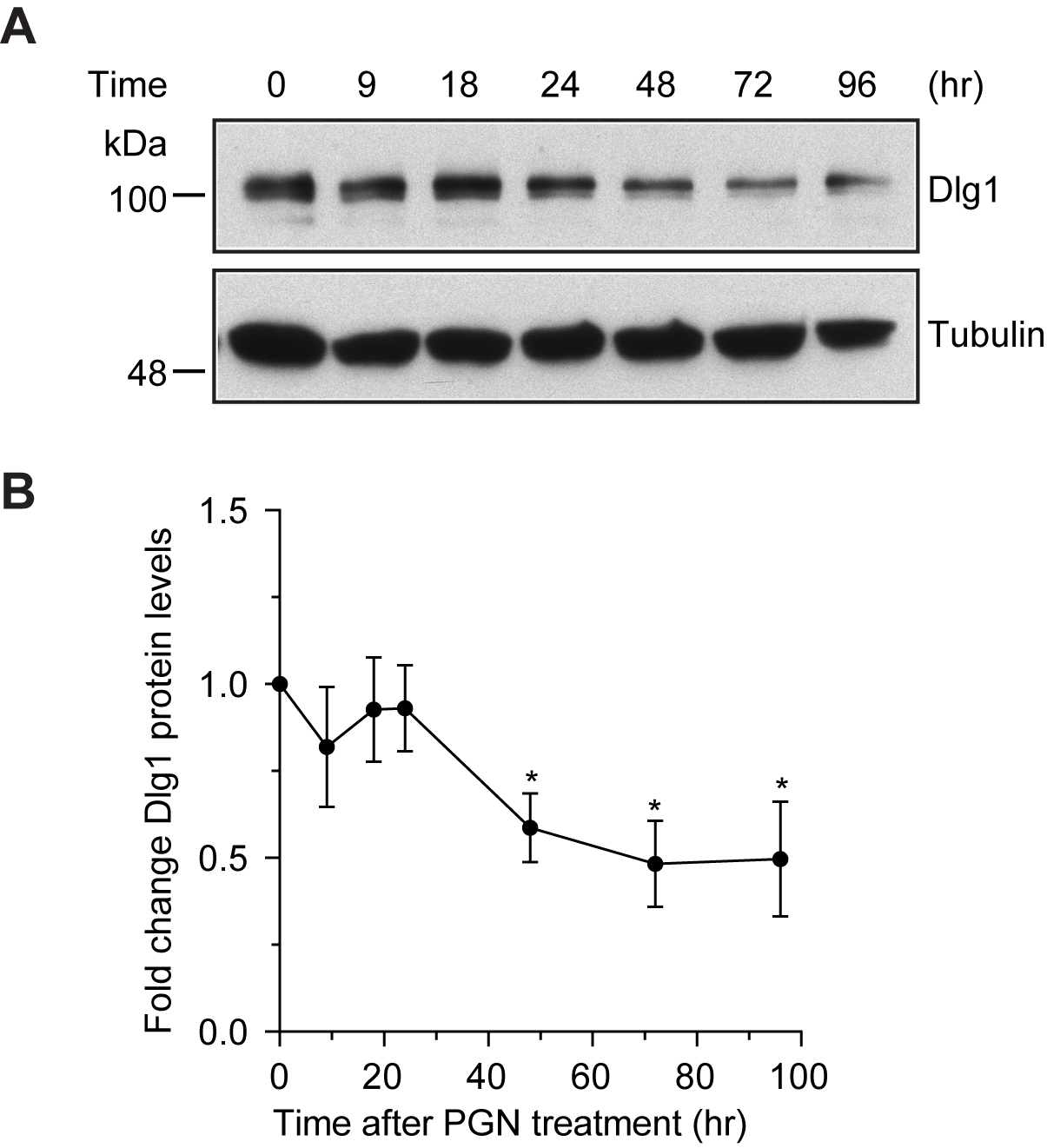

Supplement: S10 Fig — S2 cells were treated with 20-HE for 24 hrs prior to PGN treatment. Cells were harvested at different times post PGN treatment and levels of the Dlg1 protein were measured by immunoblot (A) and quantified (B; n = 3). The Tubulin protein serves as a loading control. (TIF) [file ppat.1006034.s010.tif]

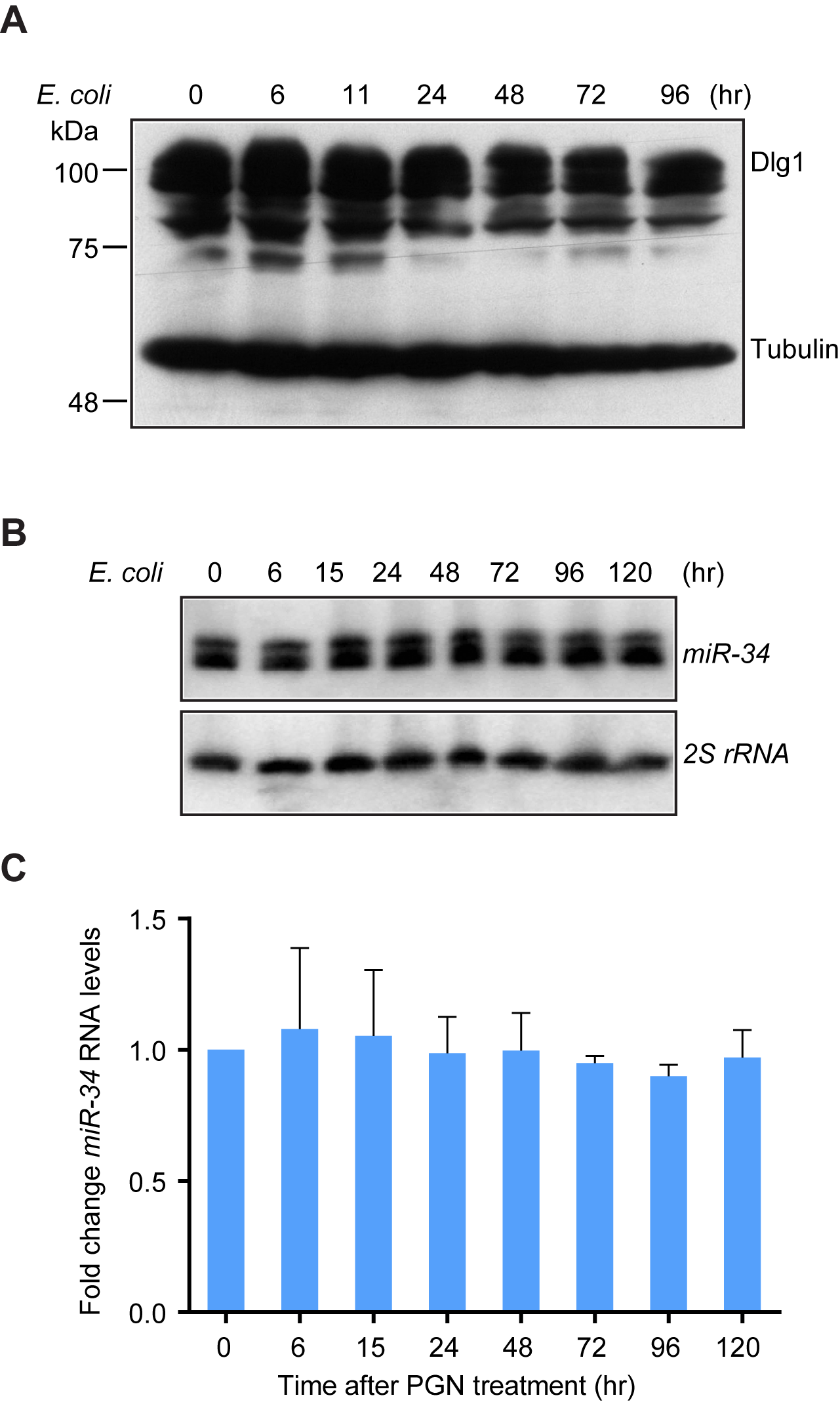

Supplement: S11 Fig — Flies were infected with E. coli using septic injury. Flies were harvested at different times and levels of the Dlg1 protein (A) and miR-34 (B) in headless fly bodies were measured by immunoblot and Northern blot, respectively. The Tubulin protein and 2S RNA serve as loading controls, respectively. (C) Levels of miR-34 were quantified and normalized against the 2S RNA (n = 3, mean + SD). (TIF) [file ppat.1006034.s011.tif]

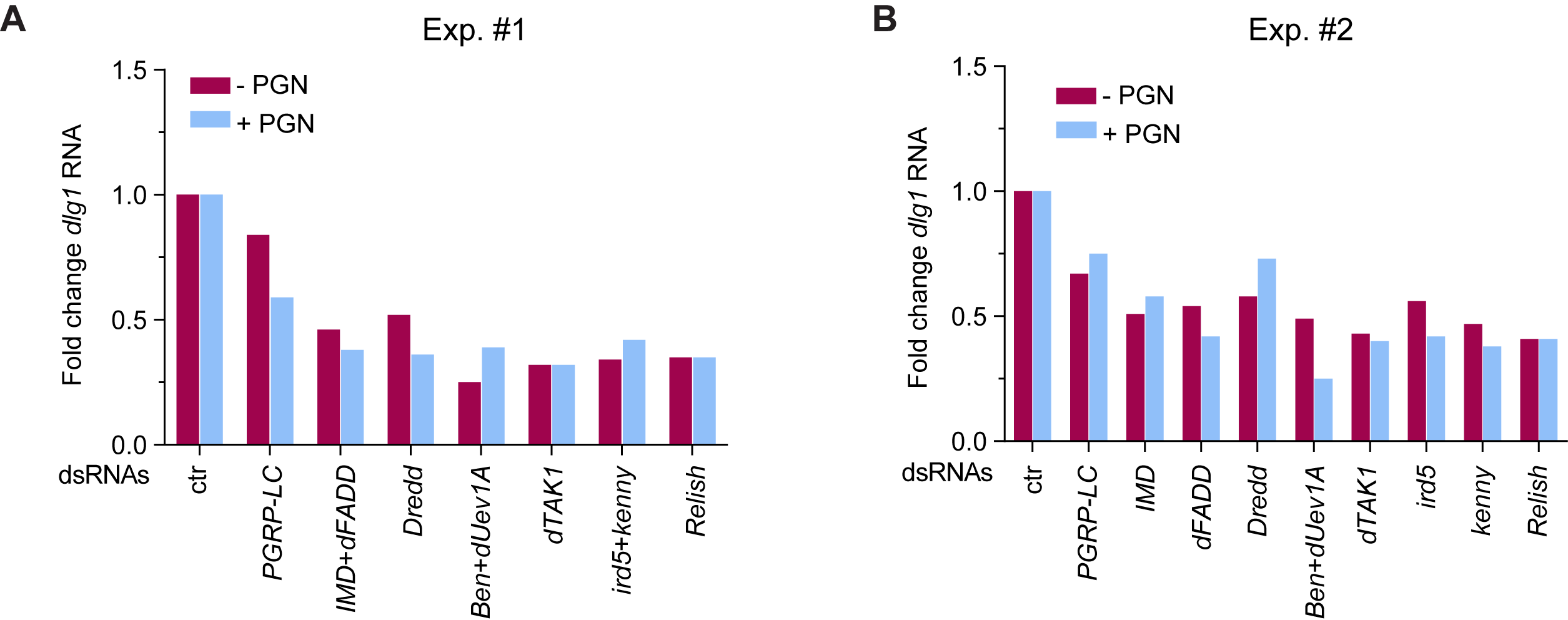

Supplement: S12 Fig — S2 cells were first treated with dsRNAs targeting various components of IMD signaling (below). Cells were subsequently treated with ecdysone for 24 hrs, and either remained untreated or treated with PGN for an additional 6 hours. Cells were harvested and levels of the dlg1 mRNAs were measured by RT-qPCR and normalized against the RpL32 mRNA. Results from two independent experiments are shown in A and B, respectively. (TIF) [file ppat.1006034.s012.tif]

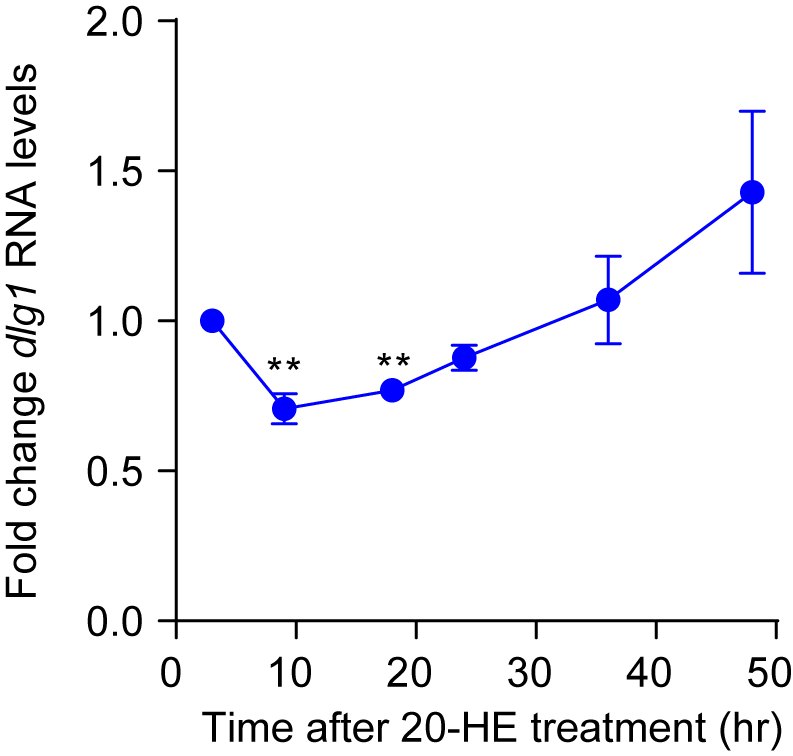

Supplement: S13 Fig — S2 cells were treated with ecdysone and harvested at different times. Levels of the dlg1 and Diptericin mRNAs were measured by RT-qPCR and normalized to the control RpL32 mRNA (n≥3). (TIF) [file ppat.1006034.s013.tif]

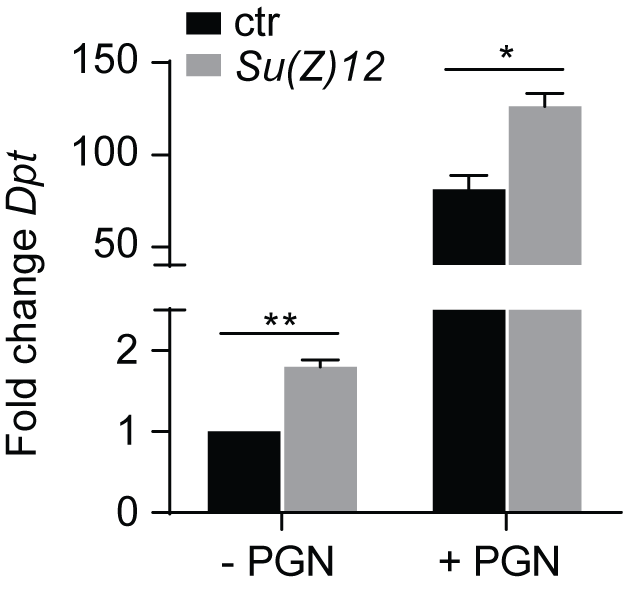

Supplement: S14 Fig — S2 cells treated with a control dsRNA or dsRNA against Suz12. Subsequently, cells were either untreated or treated with PGN. Total RNA was isolated and levels of Diptericin were measured and normalized to the RpL32 mRNA (n = 3). (TIF) [file ppat.1006034.s014.tif]
