# Supplementary material for: miR-34 Modulates Innate Immunity and Ecdysone Signaling in Drosophila
Source: PLoS Pathog. 2016 Nov 28;12(11):e1006034. doi: 10.1371/journal.ppat.1006034 (PMC5125713; doi:10.1371/journal.ppat.1006034)
Supplement: S1 Table — (PDF) [file ppat.1006034.s015.pdf]

Table S1. List of miRNAs tested in the screen.

miRNA	Fold change *diptericin* mRNA un-infected	Fold change *diptericin* mRNA *E. coli*-infected	Notes
miR-1	1.07	1.01	
miR-10	0.10	1.41	
miR-100	0.82	1.95	
miR-1000	0.17	1.27	
miR-1001	1.30	0.61	
miR-1003	9.92	2.03	
miR-1004	1.83	0.78	
miR-1006	0.12	0.77	
miR-1007	24.60	0.85	
miR-1009	N/A	0.89	
miR-1010	3.40	0.82	
miR-1011	1.54	0.49	
miR-1012	N/A	0.83	
miR-1013	0.91	0.59	
miR-1017	5.58	2.01	
miR-12	0.13	0.86	
miR-124	0.22	1.19	
miR-133	N/A	0.68	
miR-137	2.27	1.57	
miR-14	0.59	0.91	
miR-184-1	0.52	1.18	
miR-184-2	0.34	1.03	
miR-184-3	0.25	1.06	
miR-184-4	0.44	1.06	
miR-190	3.46	0.61	
miR-210	1.26	1.14	
miR-252	0.93	0.74	
miR-260	0.33	0.48	
miR-263a	0.99	0.75	
miR-263b	0.17	1.14	
miR-274	0.13	0.74	
miR-275	0.25	1.00	
miR-275-305	0.55	N/A	
miR-276a	3.58	1.18	
miR-276b	2.07	1.52	
miR-277	0.13	1.11	
miR-278	3.97	0.18	
miR-280	0.57	1.27	
miR-281-1	0.35	0.82	
miR-281-1,2	0.96	0.51	
miR-282	0.18	0.89	
miR-284	0.22	N/A	
miR-285	1.82	1.24	
miR-286	3.76	0.89	
miR-2b	0.92	1.23	
miR-303	0.28	0.60	
miR-304	0.40	0.90	
miR-305	0.17	0.95	
miR-307	10.60	1.49	
miR-308	0.11	0.99	
miR-309	1.65	0.90	

miR-31	0.93	1.48	
miR-310	1.27	0.78	
miR-311	1.90	0.68	
miR-312	0.93	0.76	
miR-318	0.90	1.19	
miR-31b	0.79	0.79	
miR-33	6.51	0.79	
miR-34	34.76	2.13	
miR-375	0.12	0.94	
miR-6-1,2,3	2.98	1.21	
miR-7	0.28	0.72	
miR-79	1.42	1.24	
miR-8	0.19	0.47	
miR-927	0.10	0.99	
miR-929	1.93	1.16	
miR-92a	16.43	0.57	
miR-92b	0.44	0.58	
miR-932	1.46	0.78	
miR-956	0.79	0.75	
miR-958	1.28	0.95	
miR-960	0.16	N/A	
miR-963	1.45	0.99	
miR-963-964	0.26	1.04	
miR-964	0.08	1.08	
miR-966	7.38	0.63	
miR-970	1.41	0.85	
miR-973	4.19	0.85	
miR-976	0.06	0.95	
miR-978	3.88	0.86	
miR-980	0.24	0.75	
miR-982	0.55	0.76	
miR-982-303	0.68	0.91	
miR-983-1	2.75	0.73	
miR-983-984	3.79	0.90	
miR-985	3.07	1.22	
miR-986	1.81	1.20	
miR-987	1.07	0.78	
miR-988	4.72	1.13	
miR-989	28.82	0.76	
miR-991	1.15	0.80	
miR-992	50.16	1.99	
miR-993	1.86	1.18	
miR-994	74.61	0.91	
miR-995	0.12	N/A	
miR-999	24.40	0.80	
miR-9a	9.21	0.54	
miR-9b	2.45	0.88	
miR-9c	0.09	0.57	
miR-iab-4	0.56	0.79	
miR-let-7	N/A	N/A	pupal lethal
			N/A; not analyzed
