# Supplementary material for: miR-34 Modulates Innate Immunity and Ecdysone Signaling in Drosophila
Source: PLoS Pathog. 2016 Nov 28;12(11):e1006034. doi: 10.1371/journal.ppat.1006034 (PMC5125713; doi:10.1371/journal.ppat.1006034)
Supplement: S2 Table — Cells were transfected with various dsRNAs as indicated. After 3 days, cells were treated with ecdysone (20-HE) at 1 μM for an additional 24 hrs. Cells were harvested, total RNA was extracted and levels of Diptericin mRNA was measured by qPCR and normalized to RpL32. Cells transfected with a dsRNA against the firefly luciferase serve as a baseline control. Also shown are fold changes in the corresponding mRNA levels upon miR-34 overexpression. (PDF) [file ppat.1006034.s016.pdf]

Table S2. Candidate *miR-34* target genes identified by Targetscan, PicTar and RNA-seq				
**Gene ID**	**FBgn number**	**SYMBOL**	**Fold change *dipt* levels**	**Fold change in mRNA levels in response to *miR-34* overexpression**
CG8224	FBgn0011300	*babo*	0.61	0.91
CG1216	FBgn0035107	*mri*	0.98	0.78
CG6827	FBgn0013997	*Nrx-IV*	0.69	0.89
CG5109	FBgn0003044	*Pcl*	0.79	0.84
CG6647	FBgn0004363	*porin*	0.99	0.93
CG18497	FBgn0016977	*spen*	1.04	0.88
CG14869	FBgn0038341	*CG14869*	1.54	0.90
CG15309	FBgn0030183	*CG15309*	0.96	0.71
CG5408	FBgn0028978	*trbl*	1.33	0.90
CG3074	FBgn0034709	*Swim*	0.72	0.81
CG1725	FBgn0001624	*dlg1*	2.39	0.54
CG2079	FBgn0029944	*Dok*	0.85	0.84
CG8938	FBgn0010226	*GstS1*	0.70	0.53
CG3322	FBgn0002528	*LanB2*	1.14	0.79
CG9381	FBgn0037705	*mura*	3.50	0.76
CG1560	FBgn0004657	*mys*	0.94	0.83
CG11172	FBgn0030505	*NFAT*	1.03	0.89
CG3307	FBgn0011474	*pr-set7*	1.23	0.90
CG4551	FBgn0016930	*smi35A*	1.11	0.68
CG5123	FBgn0003997	*W*	0.56	0.89
CG13604	FBgn0039137	*CG13604*	0.84	0.75
CG1407	FBgn0033474	*CG1407*	0.33	0.80
CG6199	FBgn0036147	*Plod*	0.89	0.92
CG7611	FBgn0037094	*CG7611*	1.28	0.80
CG7781	FBgn0032021	*CG7781*	0.96	0.52
CG8468	FBgn0033913	*CG8468*	3.02	0.74
CG32180	FBgn0000567	*Eip74EF*	1.44	0.76
