# Supplementary material for: miR-34 Modulates Innate Immunity and Ecdysone Signaling in Drosophila
Source: PLoS Pathog. 2016 Nov 28;12(11):e1006034. doi: 10.1371/journal.ppat.1006034 (PMC5125713; doi:10.1371/journal.ppat.1006034)
Supplement: S3 Table — (PDF) [file ppat.1006034.s017.pdf]

Table S3. Oligo nucleotides used in this study.

RT-qPCR of pri-miRNAs	Sequence	efficiency (%)
miR-34-3-F	TCACATCGGATTGTCTGGAA	101
miR-34-3-R	GCGTCTGGTTGAGCAGTTG	
pri-miR-277-F	CCGCTGGCTAAGGTTTATCA	91
pri-miR-277-R	AACAAAAGTGCGGTGGAAAC	
pri-miR-317-F	TAGTGCTCCCGATTTGTGTG	93
pri-miR-317-R	CGAACTGGAAAATGCCGTAT	
pri-bantam-F	AAACGCTCAGATGCAGATGTTG	90
pri-bantam-R	AGTCAAACCAAATCGAAAACcG	
RT-qPCR of mRNAs		
Dipt-F	TTGCAGTCCAGGGTCACCAG	104
Dipt-R	TTCAGTCCAATCTCGTGGcG	
Drosomycin-F	GCCCTCTTCGCTGTCCTGAT	97
Drosomycin-R	AGGGACCCTTGTATCTTCcG	
Cecropin A1-F	AAGCTGGGTGGCTGAAGAAA	106
Cecropin A1-R	TGTTGAGCGATTCCCAGTCC	
Attacin A-F	CACAACTGGCGGAACTTTGG	95
Attacin A-R	AAACATCCTTCACTCCGGGC	
Defensin-F	TGTCCTGGTGCATGAGGATG	93
Defensin-R	AGTTCCACTTGGAGAGTAGGTC	
Metchnikowin-F	GCTACATCAGTGCTGGCAGA	101
Metchnikowin-R	TTAGGATTGAAGGGCGACGG	
Pirk-F	AGAGCACGAGCAGGGTAAATC	96
Pirk-R	TGTTGTTCTCAATGCGGTACTC	
Eip75B-F	CAATCACAATCAGGTGGTGC	91
Eip75B-R	CTCCACAATCTGTCGCTTCA	
Eip74EF-F	CGAAACTTTCCCCAAAACAA	92
Eip74EF-R	ACTGCTTAAACGAGGGCTCA	
dlg1-F	ACCTGGAGAACGTAACGCAC	66
dlg1-R	ATGCACCTGACTTTGGCTCT	
RpL32-F	ATCGGTTACGGATCGAACAA	102
RpL32-R	GACAATCTCCTTGCGCTTCT	
BRC-F	TAACCTCGGCGTTCGAGAATC	111
BRC-R	TTTGCAGGGTGTGCTCTTGA	
EcR-F	GCAAGGGGTTCTTTCGACG	93
EcR-R	CGGCCAGGCACTTTTTCAG	
FMR-F	CACAACCACACCACCGAAGAG	103
FMR-R	TTACTGTCCGCTCTCTCCAcG	
Srp-F	CATGCGGCCTGTACTATAAGC	94
Srp-R	CTCGCTCTTCGTTCCTTTCGG	
Twi-F	AAGTCCCTGCAGCAGATCAT	93
Twi-R	CGGCACAGGAAGTCAATGTA	
ap-F	ACACGCCACTGACAAAGGG	91
ap-R	GAGGAGTCGTTGTGGGGTG	
Drosha-F	GAAGACCAAAGATCGTGCcG	105
Drosha-R	CACAATACAGGAGACCCTTGTCCA	
Northern blotting		
Probe-miR-9a-5p	TCATACAGCTAGATAACCAAAGA	
Probe-miR-92a-3p	ATAGGCCGGGACAAGTGCAATG	
Probe-miR-34-5p	CACAACCAGCTAACCACACTGCCA	
Probe-2S rRNA	TACAACCCTCAACCATATGTAGTCCAAGCA	
dsRNA synthesis		
T7-BRC-25765-F	TAATACGACTCACTATAGGGCCCTGGTGGAGTTCATCTA	
T7-BRC-25765-R	TAATACGACTCACTATAGGGAGCAGCTGGTTGTTGATGTG	
T7-EcR-04910-F	TAATACGACTCACTATAGGGTGCTCGTCGGAGGTGA	
T7-EcR-04910-R	TAATACGACTCACTATAGGGTTCTCCTCCTGGGTAATCTG	
T7-Dlg1-29546-F	TAATACGACTCACTATAGGGGAATGGCGATGATAGCTGGT	
T7-Dlg1-29546-R	TAATACGACTCACTATAGGGTGCAGCTTAACAACATTGCC	
T7-CG8468-33913-F	TAATACGACTCACTATAGGGCTACGTGGCGTTCGTATCCT	
T7-CG8468-33913-R	TAATACGACTCACTATAGGGAGCAGGATTTTGGTCAGTGG	
T7-mura-37705-F	TAATACGACTCACTATAGGGGCACTTTGGAAACTCCGAAC	
T7-mura-37705-R	TAATACGACTCACTATAGGGGAGGGCAGTTCACCCATAAG	
T7-Srp-29336-F	TAATACGACTCACTATAGGGTCTTGGGTCAACATGAGCAG	
T7-Srp-29336-R	TAATACGACTCACTATAGGGTCGATTTTATGCTGTTGGCA	
T7-Twi-04720-F	TAATACGACTCACTATAGGGCCAAACCGGGGGATCACT	
T7-Twi-04720-R	TAATACGACTCACTATAGGGCAAACTGCCGGCACTG	
T7-ap-37384-F	TAATACGACTCACTATAGGGCATCTCCGTGCACAGTCCTA	
T7-ap-37384-R	TAATACGACTCACTATAGGGTAGAAACAACATGTGGGGCA	
E75-DRSC23047-F	TAATACGACTCACTATAGGGCAATCACAATCAGGTGGTGC	
E75-DRSC23047-R	TAATACGACTCACTATAGGGAATATCGCTGCGCTTCATCT	
E74-DRSC40711-F	TAATACGACTCACTATAGGGCTACTGCTATGGCGGAAGGA	
E74-DRSC40711-R	TAATACGACTCACTATAGGGTCTGCATCTGCACTGAGCTT	
Plasmid Construction		
Bam-dlg1-UTR-F	GGGGATCCAGGGTAAACACTGCATCC	
SalI-dlg1-UTR-R	GCTCCTATTGTTGTTCTCG	
dlg-mut-F	CCACCACAACTTGGAGTGACGGGCCTCGAGTTCGATG	
dlg-mut-R	CATCGAACTCGAGGCCCGTCACTCCAAGTTGTGGTGG	
Bam-E75-site 1-F	GATCACAGCCAACAGCAGGGCACTGCTGCAGACAGCCAACAGCAGGGCACTGCTGCAGACAGCCAACAGCAGGGCACTGCTG	
Sal-E75-site 1-R	TCGACAGCAGTGCCCTGCTGTTGGCTGTCTGCAGCAGTGCCCTGCTGTTGGCTGTCTGCAGCAGTGCCCTGCTGTTGGCTGT	
Bam-E75-site 2-F	GATCGCCGCCTGAAAAAGTGCATTGCCGCAGGCCGCCTGAAAAAGTGCATTGCCGCAGGCCGCCTGAAAAAGTGCATTGCCG	
Sal-E75-site 2-R	TCGACGGCAATGCACTTTTTCAGGCGGCCTGCGGCAATGCACTTTTTCAGGCGGCCTGCGGCAATGCACTTTTTCAGGCGGC	
Bam-E75-site 3-F	GATCAGGGCTGCCTGCAGTACATTGTCGCAGAGGGCTGCCTGCAGTACATTGTCGCAGAGGGCTGCCTGCAGTACATTGTCG	
Sal-E75-site 3-R	TCGACGACAATGTACTGCAGGCAGCCCTCTGCGACAATGTACTGCAGGCAGCCCTCTGCGACAATGTACTGCAGGCAGCCCT	
Bam-E75-site 4-F	GATCAGCAGCAGCTCCACACCGCCGCAGCAGCAGCAGCTCCACACCGCCGCAGCAGCAGCAGCTCCACACCGCCG	
Sal-E75-site 4-R	TCGACGGCGGTGTGGAGCTGCTGCTGCGGCGGTGTGGAGCTGCTGCTGCGGCGGTGTGGAGCTGCTGCT	
Bam-E75-site 5-F	GATCTCGGTGATCACCACCACCTGCCACAGCTCGGTGATCACCACCACCTGCCACAGCTCGGTGATCACCACCACCTGCCA	
Sal-E75-site 5-R	TCGATGGCAGGTGGTGGTGATCACCGAGCTGTGGCAGGTGGTGGTGATCACCGAGCTGTGGCAGGTGGTGGTGATCACCGA	
Bam-E75-mut 1-F	GATCACAGCCAACAGCAGGTGTGACGGTCAGACAGCCAACAGCAGGTGTGACGGTCAGACAGCCAACAGCAGGTGTGACGG	
Sal-E75-mut 1-R	TCGACCGTCACACCTGCTGTTGGCTGTCTGACCGTCACACCTGCTGTTGGCTGTCTGACCGTCACACCTGCTGTTGGCTGT	
Bam-E75-mut 2-F	GATCGCCGCCTGAAAAAGTTGTGACGGTCAGGCCGCCTGAAAAAGTTGTGACGGTCAGGCCGCCTGAAAAAGTTGTGACGG	
Sal-E75-mut 2-R	TCGACCGTCACAACTTTTTCAGGCGGCCTGACCGTCACAACTTTTTCAGGCGGCCTGACCGTCACAACTTTTTCAGGCGGC	
Bam-E75-mut 3-F	GATCAGGGCTGCCTGCAGTTGTGACGGTCAGAGGGCTGCCTGCAGTTGTGACGGTCAGAGGGCTGCCTGCAGTTGTGACGG	
Sal-E75-mut 3-R	TCGACCGTCACAACTGCAGGCAGCCCTCTGACCGTCACAACTGCAGGCAGCCCTCTGACCGTCACAACTGCAGGCAGCCCT	
Bam-E75-mut 4-F	GATCAGCAGCAGCTCCTGTGACGGTCAGCAGCAGCAGCTCCTGTGACGGTCAGCAGCAGCAGCTCCTGTGACGG	
Sal-E75-mut 4-R	TCGACCGTCACAGGAGCTGCTGCTGACCGTCACAGGAGCTGCTGCTGACCGTCACAGGAGCTGCTGCT	
Bam-E75-mut 5-F	GATCTCGGTGATCACCACTGTGACGGTCAGCTCGGTGATCACCACTGTGACGGTCAGCTCGGTGATCACCACTGTGACGG	
Sal-E75-mut 5-R	TCGACCGTCACAGTGGTGATCACCGAGCTGACCGTCACAGTGGTGATCACCGAGCTGACCGTCACAGTGGTGATCACCGA	
EcoR-miR-34-F	GGAATTCGCGACAAAAAAGCTCCCC	
Bam-miR-34-R	GGGGATCCGATAACTACTGCCATAACC	

Sac-Dlg1-PD-F	GCCGAGCTCATGACAACGAGGAAAAAGAAGC						
Bam-Dlg1-PD-R	GGGGATCCTCATAGAGATTCCTTGGAAGG						
miR-34-p2-F-65	TTCACATCCATCCATCTCCA						
miR-34-p2-R-225	CACGATCTTGGGCGATAAAT						
miR-34-p2-F-217	AAGATCGTGTGCCAGCTCTC						
miR-34-p2-R-356	TGAAAATTCCGCTTTTCCAC						
miR-34-p2-F-480	CAGCGAAACATCACCTTGAA						
miR-34-p2-R-627	GAAACCGAAAGCTTTTGCAG						
miR-34-p2-F-657	TGAGTGCGGAGAGAGAGCTT						
miR-34-p2-R-860	CAGCTGCCACAACACACAC						
miR-34-p2-F-973	GCAAGCGAGAACAAGACACA						
miR-34-p2-R-1140	GATGGCAGCAGCTAACACTG						
miR-34-p2-m1-F	GTGTATATTTTTTTTGGGGGATTCATC						
miR-34-p2-m1-R	GGAATGTACATATGTACATACATATGTATGGAGA						
miR-34-p2-m2-F	ATTATAATGACAGCAGGTCGTGCA						
miR-34-p2-m2-R	TCGATAAAACGAGAGCTGGCA						
miR-34-p2-m3-F	TTTGTTTGCGTCATGTGCA						
miR-34-p2-m3-R	TTTATCTATTCCTTACCCTCTCTTTCGC						
miR-34-p2-m4-F	TAGTTTGACTTTTTAGTTTTTAGCCGCG						
miR-34-p2-m4-R	GAAGCGCAACTGTCAGCTG						
miR-34-p2-m5-F	CATCGCTGCTGCAGTCG						
miR-34-p2-m5-R	GCGTCGACGTCAACTGC						
